# Association of donor-specific antibodies with adverse outcomes in solid organ transplantation: A systematic review and meta-analysis of 69 studies

**DOI:** 10.3389/fimmu.2025.1633853

**Published:** 2025-09-12

**Authors:** Zhong-yu Kang, Xue-ya Han, Chun Liu, Wei Liu, Dai-hong Li

**Affiliations:** Department of Blood Transfusion, Tianjin First Central Hospital, School of Medicine, Nankai University, Tianjin, Nankai, China

**Keywords:** preformed donor-specific antibodies, solid organ transplantation, antibody-mediated rejection, graft survival, mean fluorescence intensity, risk stratification, organ-specific risk, HLA antibodies

## Abstract

**Importance:**

Preformed donor-specific antibodies (pre-DSAs) are a significant immunologic barrier in solid organ transplantation (SOT), yet their association with post-transplant outcomes lacks consensus, limiting standardized clinical management.

**Objective:**

To determine the association between pre-DSA and posttransplant complications, including antibody-mediated rejection (AMR), T cell-mediated rejection (TCMR), graft loss, and patient mortality, with subgroup analyses stratified by organ type and MFI thresholds (1,000 cutoff).

**Data sources:**

Systematic review of 3,322 studies from PubMed, Embase and the Cochrane Library (from inception to February 2024) following the PRISMA guidelines.

**Study selection:**

Sixty-nine observational studies (22,737 transplant recipients; 3,787 pre-DSAs+), including retrospective and prospective cohorts, encompassing kidney (KT) (41 studies), liver (LT) (13), lung (6), heart (3), and other organ transplants.

**Main outcomes and measures:**

Primary: AMR, TCMR, graft loss, patient death.

Secondary: Biliary complications, bacteremia, delayed graft function (DGF).

**Results:**

Pre-DSAs positivity conferred significantly elevated risks of AMR (RR = 5.21, 95%CI 4.01–6.79), graft loss (RR = 2.11, 1.72–2.60), and mortality (RR = 1.62, 1.39–1.89) compared with pre-DSAs–negative recipients, with marked heterogeneity across organ types. KTs faced the highest risk of AMR risk (RR = 6.09, 4.39–8.46), whereas LT recipients exhibited elevated mortality (RR = 1.81, 1.30–2.53) but lower AMR rates (RR = 1.81 *vs*. KT). The thoracic organs (heart/lung) had no significant association with AMR (RR1.32, 0.86–2.03). Stratification by MFI thresholds revealed amplified risks at MFI≥1,000, particularly for AMR (RR = 7.51 *vs* 4.65 at MFI<1,000; Pinteraction<0.001) and loss of graft (RR = 2.30 *vs* 1.81; P = .032). KT with MFI≥1,000 had the highest cumulative hazards (AMR: RR = 8.12, 5.94–11.10; graft loss: RR = 2.55, 1.98-3.28), whereas LT recipients with MFI≥1,000 had higher mortality RR = 2.01 (1.44–2.80). Secondary outcomes included increased delayed graft function (DGF: RR = 1.49, 1.12–1.98) in pre-DSA+ patients, driven by KT (RR = 1.82, 1.30–2.55), but no association with T-cell–mediated rejection (TCMR: RR = 1.10, 0.94–1.28).

**Conclusions:**

Pre-DSAs is a strong independent predictor of AMR and graft loss in SOT, with amplified risks in KT and cohorts with DSA+ MFI≥1,000. These findings advocate for universal pretransplant DSAs screening and DSA+MFI-guided desensitization to prioritize high-risk patients. Organ-specific strategies, intensified AMR surveillance in KTs, and mortality-focused monitoring in LTs, are critical to improving outcomes.

## Introduction

Solid organ transplantation (SOT) is the optimal treatment for patients with end-stage organ failure. Advancements in immunosuppression, clinical care, and surgical techniques have significantly improved clinical and patient outcomes (1, 2). Nonetheless, immune-mediated complications remain a significant barrier to graft survival (3–5). Preformed donor-specific anti-human leukocyte antigen (HLA) antibodies (pre-DSAs), directed against donor HLA antigens prior to transplantation, are critical mediators of post-transplant outcomes across organ types. Recently, pre-DSAs have been established as a major risk factor for antibody-mediated rejection (AMR) and long-term graft failure in kidney transplantation (KT) (6), as well as liver (LT) (7), lung (8), heart (9), and intestinal transplants (10).

The impact of pre-DSAs exhibits profound organ-specific heterogeneity, shaped by distinct immunological microenvironments and clinical practices (11–13). Pretransplant donor-specific antibodies (pre-DSAs) in kidney transplantation are consistently associated with higher risks of antibody-mediated rejection (AMR), graft loss, and accelerated decline in renal function. In a large, contemporary multicenter cohort, pre-DSAs predicted AMR and inferior graft outcomes, with cumulative MFI providing stronger risk discrimination than a single highest MFI; notably, class II pre-DSAs were linked to adverse outcomes at lower MFI ranges than class I (14). Beyond HLA class, locus-specific differences also matter: recipients transplanted in the presence of a preformed DP-DSA had a higher 2-year incidence of biopsy-proven AMR than those with Cw-DSA, and AMR risk rose with increasing MFI (15). These findings align with contemporary recommendations emphasizing the clinical utility of DSA characterization (including MFI and complement-activating properties) when contextualizing alloimmune risk (16). Quantitatively, even with a negative cell-based crossmatch, pre-DSAs detected by solid-phase assays at commonly applied thresholds (e.g., MFI ≥500) nearly double AMR risk (RR 1.98) and increase graft failure by 76% compared with DSA-negative recipients (6). Similar associations are observed across studies using a range of MFI cut-offs (300–1500).

Pre-DSAs, particularly those targeting class II HLA antigens or with high DSA MFI (although MFI cutoff thresholds varied widely across studies), are associated with increased risks of acute rejection, graft loss, and reduced patient survival in LT, especially in studies involving simultaneous liver-KTs or deceased donor grafts (7, 17–19). Conflicting evidence exists, as some studies report no significant impact of pre-DSAs on graft or patient outcomes, particularly in living donor LT, suggesting that factors like graft size, immunosuppression protocols, donor type, and inconsistent MFI thresholds may modulate the clinical relevance of pre-DSAs (20). Pre-DSAs also significantly impact outcomes of patients subjected to heart, lung, and intestinal transplantation, although their effects vary by organ type. In lung transplantation, pre-DSAs, particularly against HLA class II antigens, are associated with higher risk of AMR, chronic lung allograft dysfunction, and mortality, with worse outcomes observed for high MFI or complement-fixing antibodies (21, 22).

In heart transplantation, pre-existing sensitization remains a key barrier to access and post-transplant outcomes. Recent multicenter data show that transplantation across pre-DSAs can be achieved with perioperative desensitization and acceptable short-term survival, though careful selection and immunomodulation are critical (23). The latest ISHLT consensus summary underscores evolving practice patterns in antibody assessment and management across the pre- and post-transplant continuum (24). Importantly, patients with pathologic AMR plus DSAs—especially when accompanied by graft dysfunction—experience inferior survival, highlighting the clinical relevance of integrating immunologic status with histopathology and biomarkers in risk stratification (25). Emerging perspectives further reinforce the link between DSAs, AMR, and novel injury markers (e.g., donor-derived cell-free DNA), supporting more precise monitoring strategies (26). For heart transplantation, pre-DSAs, especially those activating complement (e.g., C3d+), correlate with increased AMR, cardiac allograft vasculopathy, and graft loss, although low/moderate levels of MFI can be managed with desensitization protocols (9, 27).

In intestinal transplantation, the presence of pre-DSAs heighten risks of early graft failure and chronic rejection, particularly in liver-free allografts, whereas liver inclusion demonstrates immunomodulatory benefits by promoting DSA clearance and reducing rejection (28, 29). These organ-specific risks underscore the need for tailored strategies to mitigate pre-DSA-related complications. Despite evidence supporting the adverse effects of pre-DSAs in organ transplants, inconsistencies in study designs, variable antibody detection thresholds, and organ-specific immunological complexities necessitate a comprehensive meta-analysis to clarify their overall clinical impact and guide standardized management strategies.

This study aims to synthesize existing evidence to comprehensively evaluate the impact of preformed DSAs (pre-DSAs) on graft survival, rejection rates (including antibody-mediated and cellular rejection), mortality, and other clinically relevant outcomes (e.g., chronic allograft dysfunction, infection risks) across solid organ transplants, including heart, lung, liver, kidney, pancreas, and intestinal transplantation, and to explore heterogeneity in outcomes based on antibody characteristics (MFI cutoff) and organ-specific immunological vulnerabilities.

## Methods

We conducted a systematic review and meta-analysis according to the Preferred Reporting Items for Systematic Reviews and Meta-Analyses (PRISMA) reporting guidelines. The protocol of the present study was not prospectively registered in international prospective systematic reviews registers (PROSPERO) or published previously. A comprehensive search strategy is detailed in the **Supplementary Materials**.

### Data sources and search strategy

We conducted a systematic literature search of PubMed, Embase, and the Cochrane Library from the inception of each database to 1 March 2025, using standardized search strategies developed in consultation with a medical librarian. The reference lists of all included studies and relevant systematic reviews were manually searched to identify potentially relevant publications. All identified records were evaluated against predetermined eligibility criteria through a two-phase screening process involving two independent reviewers (K.Z.Y. and H.X.Y.). Disagreements at both stages were resolved through discussion with a senior investigator (L.D.H.). The search using a combination of keywords specified the focus of each study: “liver transplantation,” “liver-kidney transplantation,” “kidney transplantation,” “lung transplantation,” “heart transplantation,” “intestinal transplantation,” “organ allocation,” “ donor-specific antibodies,” and “ preformed.” The complete search syntax for each database is provided in **Supplementary Table 1**. No language or publication status restrictions were applied.

### Outcomes

Prespecified endpoints included AMR, TCMR, graft loss (re-transplantation or organ-specific functional failure) and all-cause mortality. Secondary outcomes included biliary complications, bacteremia, delayed graft function (DGF), cytomegalovirus (CMV) infection, and composite rejection events. We extracted the results using standardized case report forms from the included studies. Subgroup analyses were stratified by transplant organ type (e.g., kidney, liver, heart, lung, and other organs), and MFI cutoff thresholds for DSAs (MFI≥1,000 *vs*. <1,000). Outcomes lacking consensus diagnostic criteria in source studies were excluded from the meta-analysis to ensure methodological rigor.

### Study selection

The study selection process was conducted by two independent reviewers (K.Z.Y. and H.X.Y.) using EndNote X9 citation management software (Clarivate Analytics), following PRISMA 2020 guidelines. During the initial screening phase, titles and abstracts were evaluated for original English-language studies using analytical study designs (randomized controlled trials, prospective/retrospective cohort studies, case-control studies) involving adult or pediatric recipients of kidney, liver, heart, lung, or other solid organ transplants with pretransplant DSA (pre-DSA) assessment. Full text evaluation of potentially eligible studies was performed to confirm the inclusion of original clinical research with a predefined clinical outcome, including AMR, graft loss, mortality, and other transplant-related outcomes. We systematically excluded studies involving nonsolid organ transplants (e.g., bone marrow, pancreatic islet cells), animal models, nonanalytic publications (editorials, letters, conference abstracts without peer-reviewed full texts), clinical studies with inaccessible full texts, case series with fewer than 10 participants, and research lacking essential outcome metrics or containing critical methodological flaws (e.g., non-comparative designs, inadequate control groups, undocumented data of pre-DSAs). Discrepancies or uncertainties were resolved by discussion with a third author (L.D.H.).

### Data extraction and quality assessment

Two independent reviewers (K.Z.Y. and H.X.Y.) systematically extracted data from the included study using a developed standardized electronic form. The extraction template captured critical variables including study identification (first author, publication year, country), methodological characteristics (study design, inclusion/exclusion criteria, data source), population demographics (age range, sex distribution), DSA status before transplantation (pre-DSA) (qualitative assessment and MFI cut-off thresholds for positivity), temporal parameters (study period enrolment dates), rationale for sample size determination and dichotomous outcome metrics (presence/absence of complications defined by the protocol). Discrepancies in data interpretation were resolved through iterative re-evaluation and final arbitration by a transplantation immunology methodologist (L.D.H.).

Two independent investigators (K.Z.Y. and H.X.Y.) evaluated the quality of observational studies using the Newcastle-Ottawa Scale (NOS): cohort selection (0–4 stars), comparability (0–2 stars), and outcome validity (0–3 stars), with studies scoring 7/9 considered of high methodological quality.

### Statistical analysis

Risk ratios (RR) with the corresponding 95% confidence intervals (95%CI) were the principal effect measures and derived through comprehensive statistical analysis using Stata v.18.0 (StataCorp). To evaluate the heterogeneity of the study, we implemented a dual-assessment approach: Cochran’s Q test (employing a significance threshold of P<.10 to indicate heterogeneity) complemented by quantification through the *I²* statistic. *I²* values were interpreted using conventional thresholds (<25% low, 25–75% moderate, >75% high heterogeneity), with values >50% considered indicative of substantial heterogeneity. Based on these assessments, we employed random effects models when significant heterogeneity was detected; otherwise, fixed effects models were applied in sensitivity analyses. Publication bias was systematically evaluated using Egger’s and Begg’s tests (with P<.10 denoting significant bias). Prespecified subgroup analyses were conducted according to organ type and MFI) cutoff values. All statistical inferences were performed using two-tailed tests, maintaining a nominal significance level of α=0.05 throughout the analysis, unless explicitly stated otherwise.

## Results

### Literature search and study characteristics

**Figure 1:**
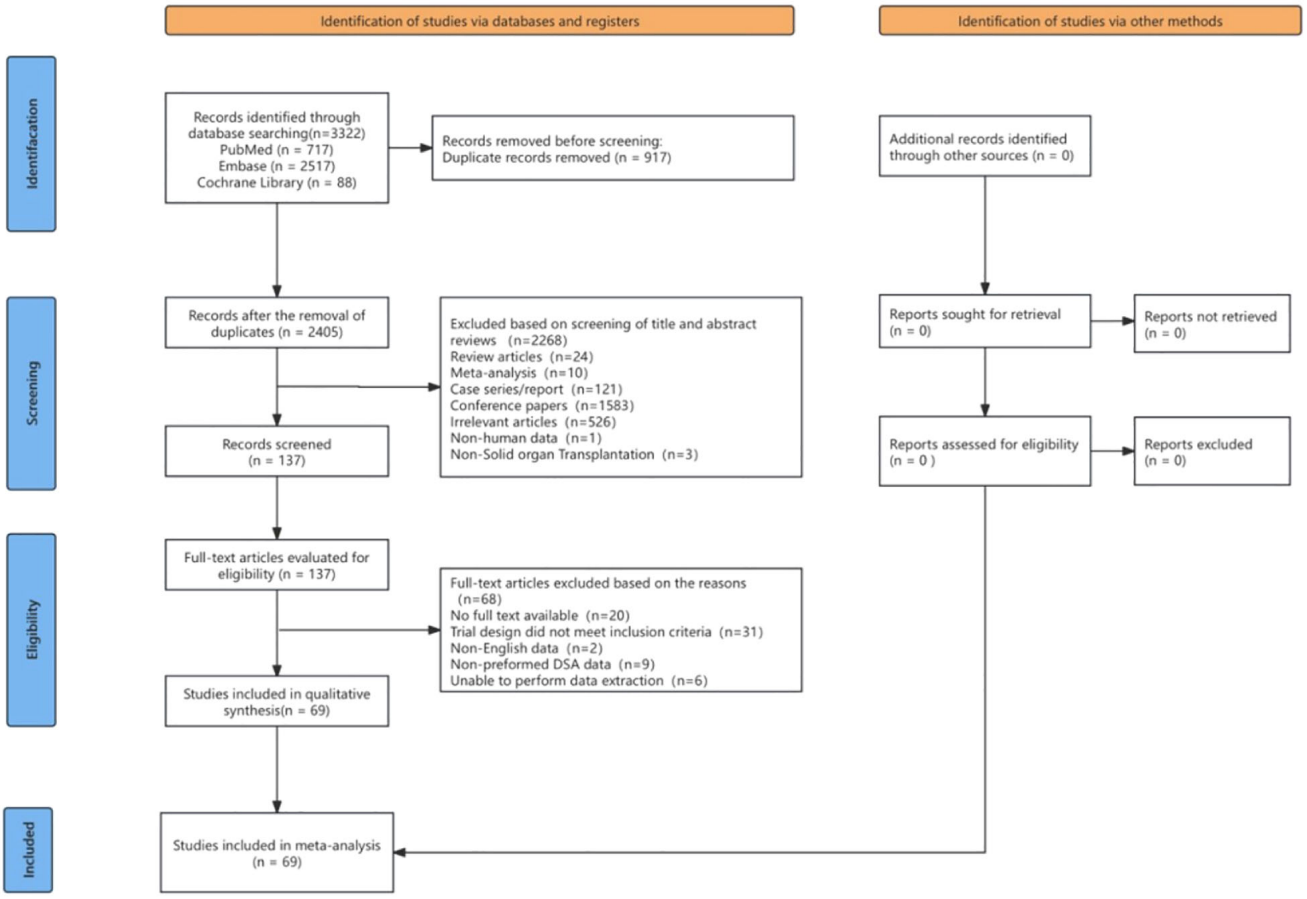
Selection process of included studies.

**Figure 1** details the study selection process. Systematic searches of PubMed, Embase, and the Cochrane Library identified 3,322 records, with no additional studies from manual reference screening. After removing 917 duplicates, 2,405 studies underwent title/abstract selection, excluding 2,268 records unrelated to pre-DSAs in SOT. Full-text review of 137 articles led to the exclusion of 68 studies for not meeting eligibility criteria (e.g., no full text available, missing pre-DSA data, or unable to perform data extraction). The final meta-analysis included 69 observational studies comprising 22,737 transplant recipients, with organ-specific cohorts as follows: KT (15,515; 68.3%), liver (3,583; 15.8%), lung (2,151; 9.5%), heart (879; 3.9%), intestinal (288; 1.3%), and combined liver-KTs (321; 1.4%).

Geographically, the studies covered 15 countries on four continents, the majority originating in the United States (n=15), Spain (n=7) and France (n=7). Other contributors included Germany (n=6), South Korea (n=6), the United Kingdom (n=5), Switzerland (n=4), Japan (n=4), and Brazil (n=2), along with single-study reports from Thailand, Pakistan, the Netherlands, Mexico, Malaysia, Poland, Portugal, Belgium, and Australia. The publication dates ranged from 2001 (the earliest database record) to 1 March 2025, capturing evolving DSA monitoring practices over two decades. The characteristics of the study and the outcomes are summarized in **Table 1**.

**Table 1:** Characteristics of all studies included in the systematic review.

No	Study (year)	Location	Duration	Data source	Population	Solid organ type	Sample size	Group	Age	Sex M/F	Outcomes	MFI cutoff	Quality score
1	Kim 2024 (50)	Korea	2015.1-2020.12	Single center	Adult	Liver	88	pre-DSA+:44 pre-DSA-:44	54.8 ± 11.3	22/66	TCMR, AMR, BCs, CMV	5000	6
2	Shizuku 2023 (51)	Japan	2014.10-2021.12	Single center	Adult	Liver	120	pre-DSA+:23 pre-DSA-:97	NR	53/67	TCMR, bacteremia, CMV, patient death	1000	7
3	Ogawa 2023 (52)	Japan	2016.1-2022.3	Single center	Adult	Liver	30	pre-DSA+:9 pre-DSA-:21	54 (24-70)	14/16	TCMR, AMR, BCs, bacteremia, CMV, patient death	1000	7
4	Goto 2022 (7)	Japan	1997.7-2016.1	Single center	Adult	Liver	150	pre-DSA+:27 pre-DSA-:123	NR	82/68	Graft loss	1000	8
5	Schluckebier 2019 (53)	Switzerland	2005.1.1-2017.12.31	Single center	Pediatric	Liver	60	pre-DSA+:20 pre-DSA-:43	NR	37/26	TCMR	1000	7
6	Legaz 2020 (54)	Spain	1998-2014	Single center	Adult	Liver	810	pre-DSA+:26 pre-DSA-:784	56.1 ± 12.1	478/332	Rejection, graft loss	1500	8
7	Del Bello 2020 (55)	France	2001.9-2018.7	Single center	Adult	Liver	284	pre-DSA+:142 pre-DSA-:142	54.5 ± 10.1	106/178	TCMR, AMR, CMV, patient death	1000	7
8	Tamura 2019 (19)	Japan	2001.8-2015.7	Single center	Adult	Liver	40	pre-DSA+:8 pre-DSA-:32	57.5 (26-66)	21/19	TCMR, CMV, rejection, graft loss, patient death	1000	7
9	Koch 2018 (56)	Germany	2008.5-2014.9	Single center	Adult	Liver	91	pre-DSA+:55 pre-DSA-:36	55.3 ± 10.4	58/33	CMV, graft loss	1500	8
10	Kim 2018 (57)	Korea	2014-2015	Single center	Adult	Liver	61	pre-DSA+:15 pre-DSA-:46	55.2 ± 7.2	45/16	TCMR, BCs, CMV	1000	6
11	O’Leary 2014 (58)	USA	2001.1.1-2009.5.1	Single center	Adult	Liver	467	pre-DSA+:86 pre-DSA-:381	NR	339/128	CMV, rejection, patient death	5000	7
12	O’Leary 2013 (59)	USA	2001.1.1-2009.5.31	Single center	Adult	Liver	1270	pre-DSA+:184 pre-DSA:1086	NR	828/442	CMV, rejection, patient death	5000	7
13	Musat 2013 (60)	USA	2009.5.1-2010.10.28	Single center	Adult	Liver	109	pre-DSA+:74 pre-DSA-:35	54.4 ± 9.8	78/31	TCMR	300	8
14	Lee 2024 (61)	Korea	2014.1-2020.12	Multi center	Adult	Kidney	2019	pre-DSA+:137 pre-DSA-:1782	47.9 ± 12.1	1191/828	TCMR, AMR, bacteremia, rejection, patient death	1000	6
15	Vilela 2023 (62)	Portugal	2017.1-2021.12	Single center	Adult	Kidney	75	pre-DSA+:15 pre-DSA-:60	55.8 ± 10.0	51/24	AMR, rejection	NR	7
16	Lee 2023 (63)	Korea	2010-2018	Single center	Adult	Kidney	1027	pre-DSA+:89 pre-DSA-:938	47.5 ± 11.3	612/415	AMR, Bacteremia, CMV, graft loss	1000	7
17	Gniewkiewicz 2023 (64)	Poland	2018-2020	Single center	Adult	Kidney	98	pre-DSA+:18 pre-DSA-:80	NR	62/36	AMR, graft loss, patient death	500	8
18	de Rougemont 2023 (65)	Switzerland	2008.5-2017.12	Multi center	Adult	Kidney	2215	pre-DSA+:411 pre-DSA-:1804	NR	1409/806	TCMR, AMR, graft loss	500	6
19	Abbas 2023 (66)	Pakistan	2015.1-2021.12	Single center	Adult	Kidney	90	pre-DSA+:45 pre-DSA-:45	27.3 ± 8.7	69/21	TCMR, AMR, rejection	1000	7
20	Seitz 2022 (67)	UK	2013.1-2020.2	Single center	Adult	Kidney	69	pre-DSA+:23 pre-DSA-:46	45.7 ± 14.1	44/25	AMR	2000	7
21	Olszowska-Zaremba 2022 (68)	Poland	2014-2018	Single center	Adult	Kidney	35	pre-DSA+:18 pre-DSA-:17	NR	24/11	TCMR, graft loss	5000	8
22	Heinemann 2022 (69)	Germany	2011.1-2012.12	Single center	Adult	Kidney	191	pre-DSA+:40 pre-DSA-:151	NR	112/79	TCMR, AMR	1000	6
23	Parajuli 2021 (70)	USA	2013.1-2017.5	Single center	Adult	Kidney	850	pre-DSA+:68 pre-DSA-:782	52.9 ± 12.9	564/286	TCMR, AMR, bacteremia, DGF, CMV, rejection, graft loss	1000	7
24	Schutt 2020 (71)	USA	2012.1.1-2015.7.8	Single center	Adult	Kidney	161	pre-DSA+:15 pre-DSA-:146	53.9 ± 13.4	90/71	TCMR, AMR, DGF, rejection, graft loss, patient death	NR	7
25	Jalalonmuhali 2020 (72)	Malaysia	2016.8-2018.6	Single center	Adult	Kidney	40	pre-DSA+:20 pre-DSA-:20	39.0 ± 11.9	23/17	Rejection	500	6
26	Uffing 2019 (73)	USA	2007.8-2015.2	Single center	Adult	Kidney	179	pre-DSA+:31 pre-DSA-:148	53.8 ± 13.4	114/65	DGF, rejection, graft loss	1000	7
27	Senev 2019 (74)	Belgium	2004.3-2014.2	Single center	Adult	Kidney	924	pre-DSA+:107 pre-DSA-:817	53.6 ± 13.2	556/358	AMR	NR	6
28	Michielsen 2019 (75)	Netherlands	1995-2005	Multi center	Adult	Kidney	330	pre-DSA+:115 pre-DSA-:115	47.5 ± 13.3	134/196	Rejection, graft loss	750	6
29	Redondo-Pachón 2018 (76)	Spain	2006.8-2014.3	Single center	Adult	Kidney	370	pre-DSA+:39 pre-DSA-:331	53.7 ± 13.5	224/146	AMR, DGF, graft loss	NR	7
30	Kwon 2018 (77)	Korea	2013.1-2016.7	Single center	Adult	Kidney	575	pre-DSA+:81 pre-DSA-:494	45.7 ± 12.3	363/212	TCMR, AMR, rejection	1000	6
31	Ixtlapale-Carmona 2018 (78)	Mexico	2012.1-2015.12	Single center	Adult	Kidney	100	pre-DSA+:24 pre-DSA-:76	38.5 ± 13.5	59/41	TCMR, AMR	500	6
32	de Sousa 2018 (79)	Brazil	2012-2016	Single center	Adult	Kidney	64	pre-DSA+:21 pre-DSA-:43	45.2 ± 10.9	29/35	TCMR, AMR, DGF, CMV, graft loss	NR	7
33	Zeche 2017 (80)	Germany	2005.1-2012.12	Single center	Adult	Kidney	174	pre-DSA+:61 pre-DSA-:113	NR	114/60	TCMR, AMR, rejection	500	8
34	Malheiro 2017 (81)	Portugal	2007-2014	Single center	Adult	Kidney	624	pre-DSA+:47 pre-DSA-:577	45.2 ± 15.5	402/222	TCMR, AMR, graft loss, patient death	1000	7
35	Schaefer 2016 (82)	Germany	NR	Single center	Adult	Kidney	80	pre-DSA+:61 pre-DSA-:19	49 (19–71)	41/39	AMR	500	7
36	Salvadé 2016 (83)	Switzerland	2008.1-2014.3	Single center	Adult	Kidney	280	pre-DSA+:24 pre-DSA-:256	NR	NR	TCMR, rejection	500	8
37	Richter 2016 (84)	Germany	2002-2009	Single center	Adult	Kidney	197	pre-DSA+:69 pre-DSA-:128	51.4 ± 13.1	121/76	Graft loss	3000	6
38	Adebiyi 2016 (85)	USA	2007.9-2012.8	Single center	Adult	Kidney	660	pre-DSA+:162 pre-DSA-:498	48.6 ± 13.6	378/280	AMR	500	6
39	Redondo-Pachón 2015 (86)	Spain	2008.1-2013.3	Single center	Adult	Kidney	259	pre-DSA+:36 pre-DSA-:223	50	165/94	TCMR, AMR, rejection	1000	7
40	Visentin J 2015 (87)	France	2001.1-2008.12	Single center	Adult	Kidney	130	pre-DSA+:44 pre-DSA-:86	NR	NR	TCMR, AMR, graft loss, patient death	500	8
41	Malheiro 2015 (88)	Portugal	2007-2012	Single center	Adult	Kidney	407	pre-DSA+:40 pre-DSA-:367	44.9 ± 15.6	271/136	TCMR, AMR, DGF, rejection, graft loss	1000	7
42	Marfo 2014 (89)	USA	2009.5.1-2012.12.31	Single center	Adult	Kidney	373	pre-DSA+:66 pre-DSA-:307	207	207/166	TCMR, AMR, DGF, CMV, rejection, graft loss, patient death	1000	7
43	Fidler 2013 (90)	Australia	2003.6-2007.10	Single center	Adult	Kidney	230	pre-DSA+:37 pre-DSA-:193	46.5 ± 12.7	155/75	AMR, rejection	1000	8
44	Crespo 2013 (91)	Spain	2006.7-2011.7	Single center	Adult	Kidney	355	pre-DSA+:28 pre-DSA-:327	NR	110/245	TCMR, AMR, DGF, graft loss	2000	7
45	Berga 2012 (92)	Spain	1997.11-2006.11	Single center	Adult	Kidney	222	pre-DSA+:36 pre-DSA-:186	48.7 ± 16.5	127/95	DGF, rejection	500	6
46	David-Neto 2012 (93)	Brazil	2002-2004	Single center	Adult	Kidney	94	pre-DSA+:16 pre-DSA-:78	41.7 ± 11.9	51/43	AMR, DGF, CMV, rejection	NR	6
47	Caro-Oleas 2012 (94)	Spain	1993.1-2020.2	Single center	Adult	Kidney	785	pre-DSA+:103 pre-DSA-:682	NR	484/301	Graft loss	1500	8
48	Song 2012 (95)	Korea	2005.6-2009.5	Single center	Adult	Kidney	27	pre-DSA+:16 pre-DSA-:11	NR	NR	TCMR, AMR, rejection, graft loss	500	6
49	Thammanichanond 2012 (96)	Thailand	2003.1-2007.12	Single center	Adult	Kidney	116	pre-DSA+:28 pre-DSA-:88	43.3 ± 12.6	65/51	TCMR, AMR, DGF, Graft loss	500	7
50	Willicombe 2011 (97)	UK	2005.10-2009.10	Single center	Adult	Kidney	480	pre-DSA+:45 pre-DSA-:435	NR	315/165	TCMR, CMV, rejection, graft loss	NR	7
51	Loupy 2009 (98)	France	2002.1-2007.3	Single center	Adult	Kidney	137	pre-DSA+:54 pre-DSA-:83	47.2 ± 13.2	NR	TCMR, graft loss, patient death	300	6
52	Amico 2009 (99)	Switzerland	1991.1-2004.11	Single center	Adult	Kidney	334	pre-DSA+:67 pre-DSA-:267	51 (13–72)	212/122	TCMR, AMR, rejection	500	6
53	Phelan 2009 (100)	USA	2004.3-2005.7	Single center	Adult	Kidney	40	pre-DSA+:12 pre-DSA-:28	NR	NR	Rejection, graft loss	NR	7
54	Gupta 2008 (101)	UK	1999.1.1-2001.12.31	Single center	Adult	Kidney	99	pre-DSA+:16 pre-DSA-:83	NR	64/31	Graft loss	NR	6
55	Dekeyser 2022 (30)	France	2008.1-2018.12	Single center	Adult	Clkt	70	pre-DSA+:43 pre-DSA-:27	50.3 ± 12.3	41/29	Rejection	500	7
56	Del Bello 2020 (18)	France	2008-2017	Multi center	Adult	Clkt	166	pre-DSA+:46 pre-DSA-:120	50.7 ± 12.9	100/66	TCMR, AMR, rejection, graft loss	1000	6
57	Yazawa 2020 (102)	USA	2009-2018	Single center	Adult	Clkt	85	pre-DSA+:19 pre-DSA-:66	55.5 ± 10.1	53/35	DGF	2000	6
58	Heise 2023 (103)	Germany	2013.2-2022.5	Single center	Adult	Lung	820	pre-DSA+:62 pre-DSA-:758	NR	438/382	CMV	1000	7
59	Parquin 2021 (31)	France	2012.1-2018.3	Single center	Adult	Lung	321	pre-DSA+:105 pre-DSA-:216	41 ± 13	154/167	AMR	500	6
60	Courtwright 2019 (8)	USA	2012.10.1-2018.2.28	Single center	Adult	Lung	203	pre-DSA+:18 pre-DSA-:185	61 (52-66)	120/83	TCMR, AMR, patient death	1000	6
61	Verleden 2017 (22)	Belgium	2010.1.1-2017.4.1	Single center	Adult	Lung	362	pre-DSA+:61 pre-DSA-:302	56 (43–61)	181/182	Rejection	500	7
62	Smith 2014 (104)	UK	1991.1-2003.12	Single center	Adult	Lung	389	pre-DSA+:27 pre-DSA-:362	NR	NR	TCMR	NR	7
63	Brugie`re 2013 (105)	France	NR	Single center	Adult	Lung	56	pre-DSA+:18 pre-DSA-:38	43.6 ± 16.1	24/32	Patient death	300	8
64	Setia 2021 (106)	USA	2010-2018	Single center	Adult	Heart	623	pre-DSA+:67 pre-DSA-:556	56.3 ± 12.3	471/152	TCMR, AMR, patient death	NR	6
65	Zhang 2018 (9)	USA	2010-2013	Single center	Pediatric	Heart	176	pre-DSA+:22 pre-DSA-:154	NR	NR	AMR	1000/2000	7
66	Gandhi 2011 (27)	USA	2006.8.1-2010.1.31	Single center	Adult	Heart	80	pre-DSA+:11 pre-DSA-:69	NR	NR	TCMR, AMR	1500	7
67	McArdle 2024 (10)	UK	2007-2019	Single center	Adult	Intestinal	95	pre-DSA+:36 pre-DSA-:59	NR	48/47	Rejection	500	6
68	Talayero 2018 (107)	Spain	2002-2017	Single center	Pediatric	Intestinal	37	pre-DSA+:5 pre-DSA-:32	NR	NR	Rejection, graft loss	1000	7
69	Abu-Elmagd 2012 (29)	USA	2000.5-2010.2	Single center	Adult	Intestinal	156	pre-DSA+:49 pre-DSA-:107	NR	NR	Graft loss	1000	8

BCs, Biliary complications; AMR, antibody-mediated rejection; TCMR, T cell-mediated rejection; DGF, delayed graft function.

### Primary outcomes

**Figure 2:**
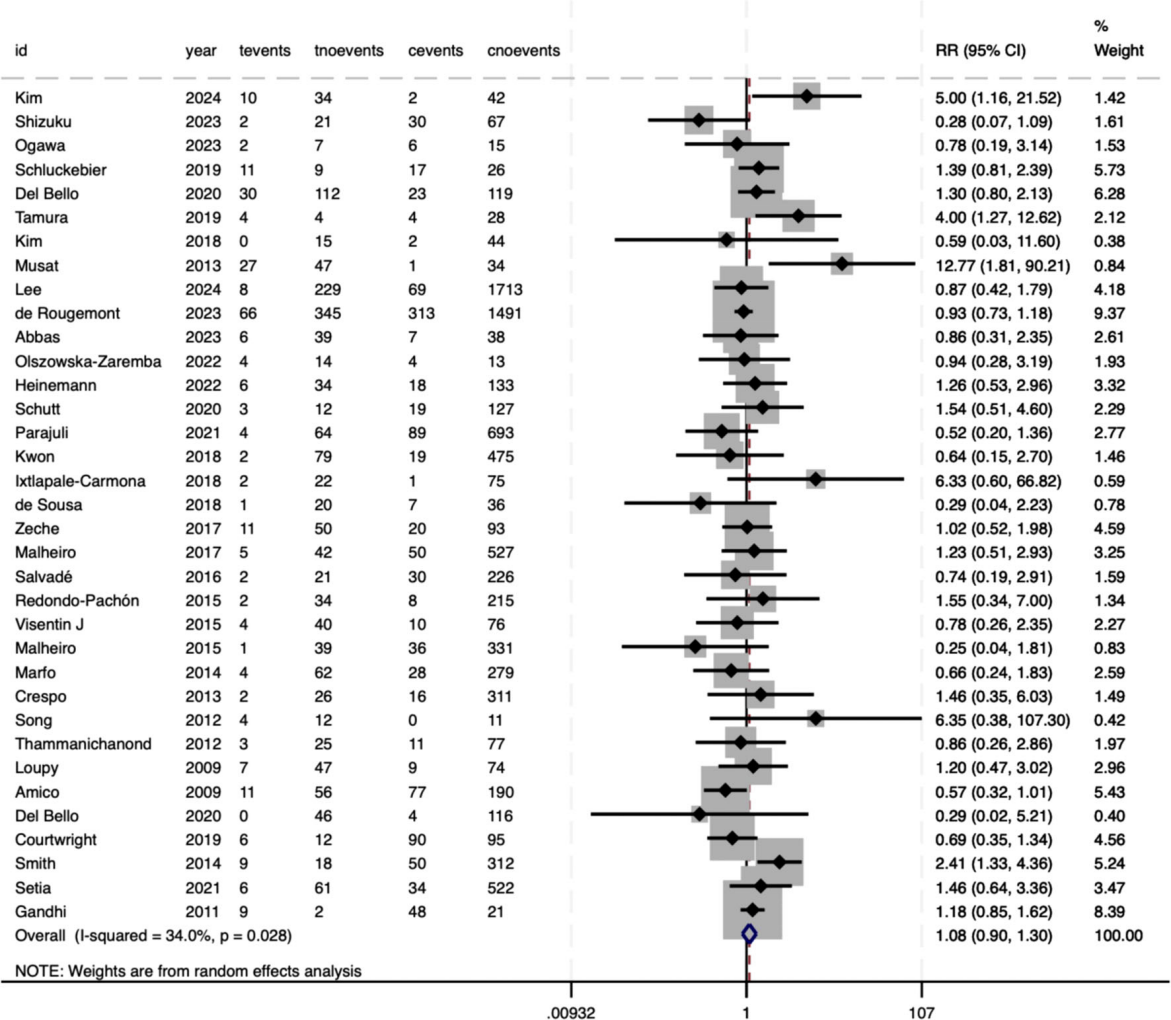
Association between pre-DSAs and the risk of TCMR.

**Figure 3:**
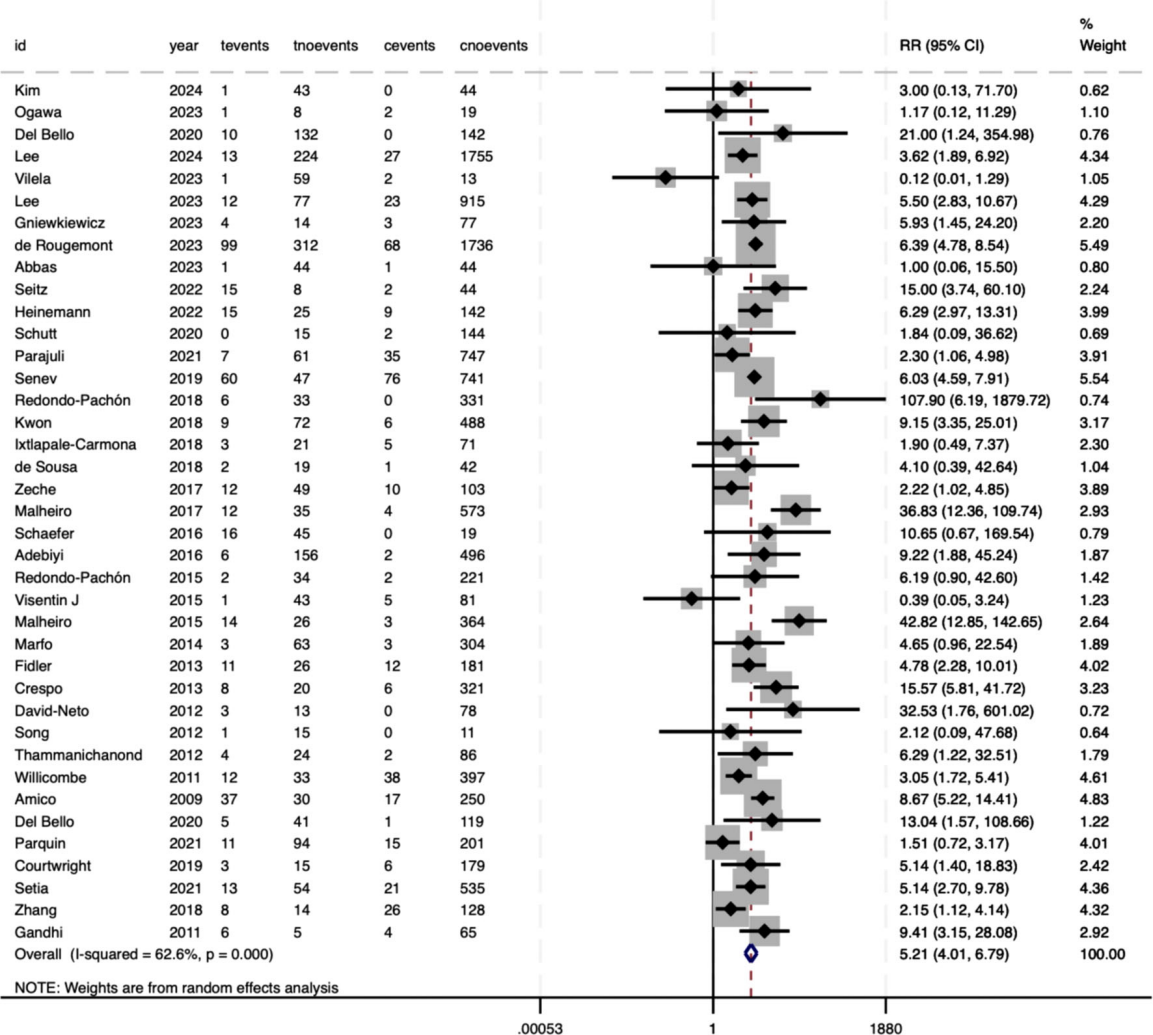
Association between pre-DSAs and the risk of AMR.

Meta-analysis revealed significant associations between pre-DSAs and adverse outcomes in SOT. For TCMR, the pooled RR was 1.08 (95%CI 0.9–1.3), which did not reach statistical significance (P = .425) (**Figure 2**). However, moderate heterogeneity was observed between studies (*I²* = 34%, P = .028), suggesting variability in effect estimates that may reflect differences in study populations or methodologies. Conversely, AMR demonstrated a robust association with pre-DSAs, with an RR of 5.21 (95%CI 4.01–6.79, P<.001), indicating a five-fold increased risk (**Figure 3**). Substantial heterogeneity (*I²* = 62.6%, P<.001) highlighted the potential clinical or methodological diversity among the included studies, which warrants a cautious interpretation of this result.

**Figure 4:**
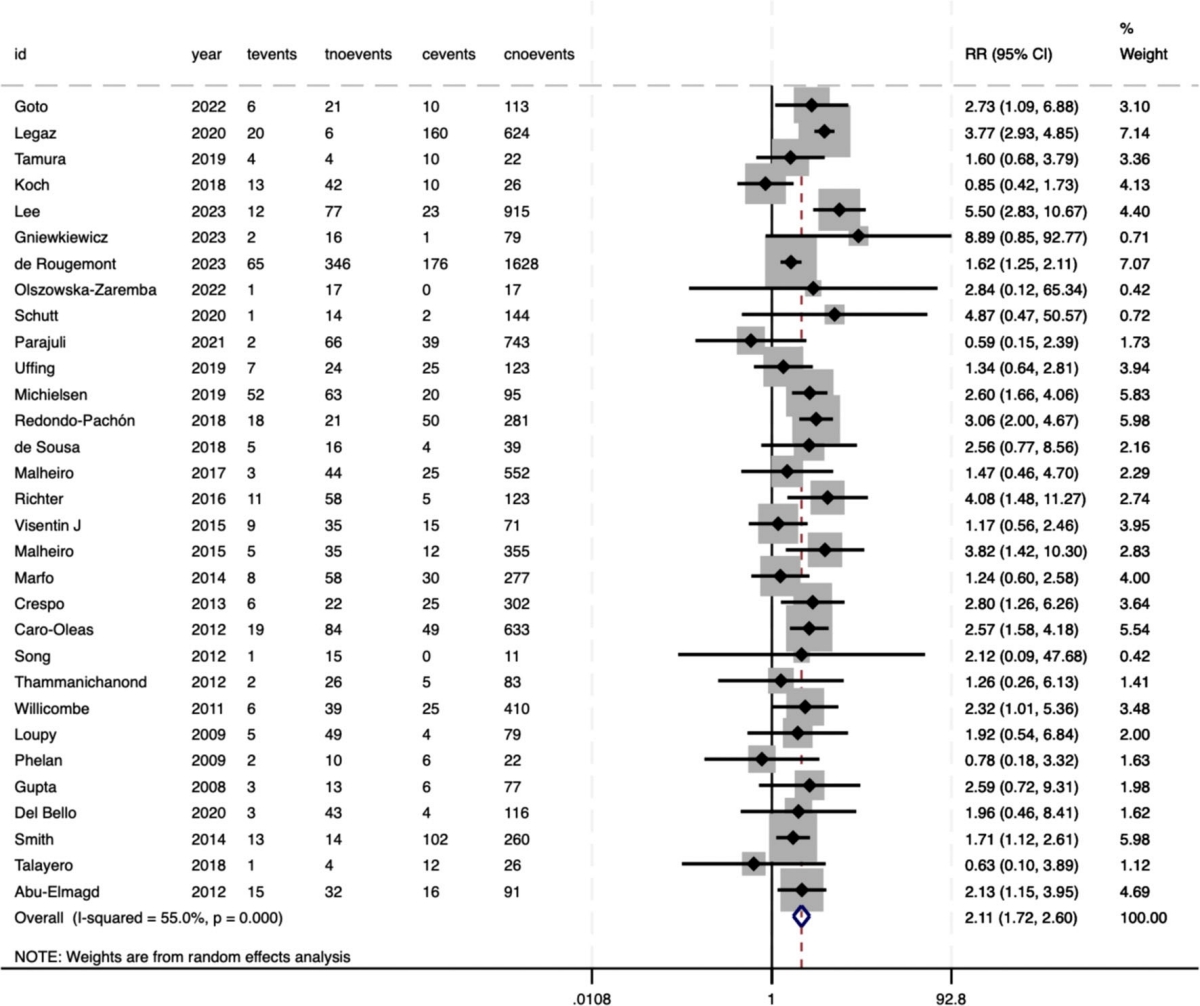
Association between pre-DSAs and the risk of graft loss.

**Figure 5:**
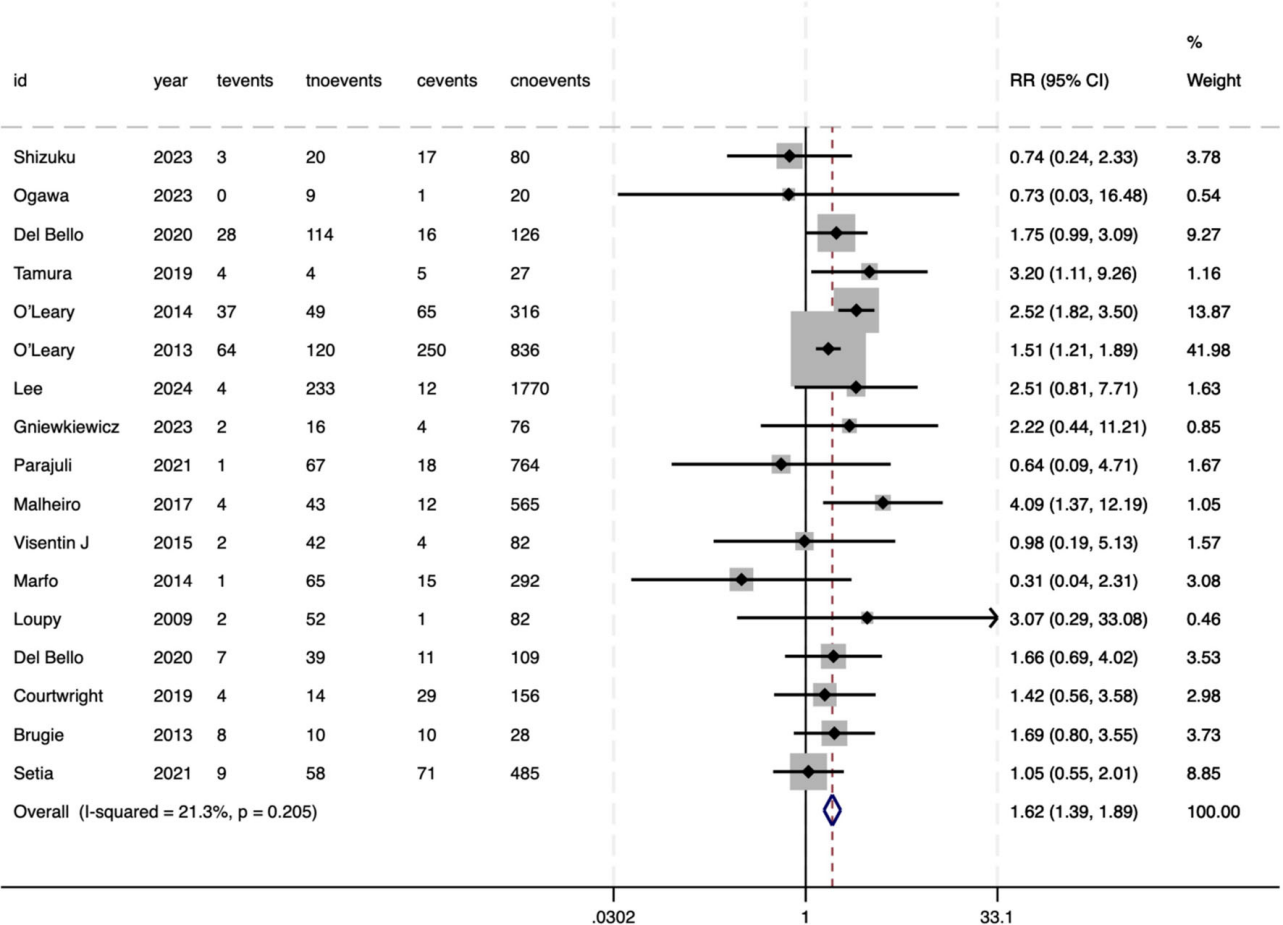
Association between pre-DSAs and the risk of patient death.

The risk of graft loss was significantly elevated in recipients with pre-DSAs (RR = 2.11, 95%CI 1.72–2.60, P<.001), with moderate heterogeneity between studies (*I²* = 55%, P<.001) (**Figure 4**). For all-cause mortality, the pooled analysis showed an RR of 1.62 (95%CI 1.39–1.89, P<.001), reflecting a 62% increased risk of death in pre-DSA-positive recipients (**Figure 5**). Although the statistical significance of mortality was clear, heterogeneity for this outcome was low (*I²* = 21.3%, P = .205), suggesting consistent effects across studies.

### Secondary outcomes

**Figure 6:**
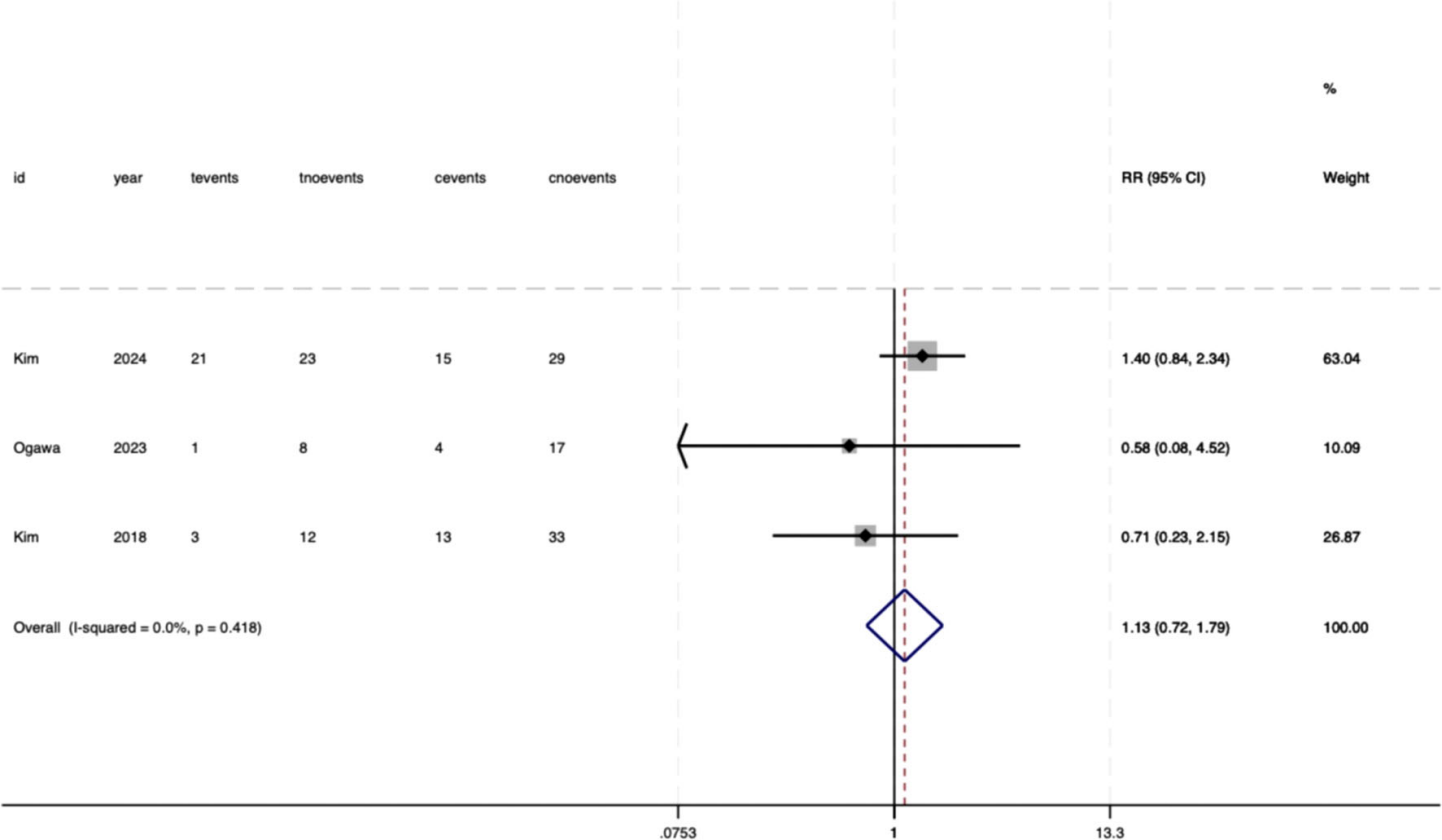
Association between pre-DSAs and the risk of biliary complications.

**Figure 7:**
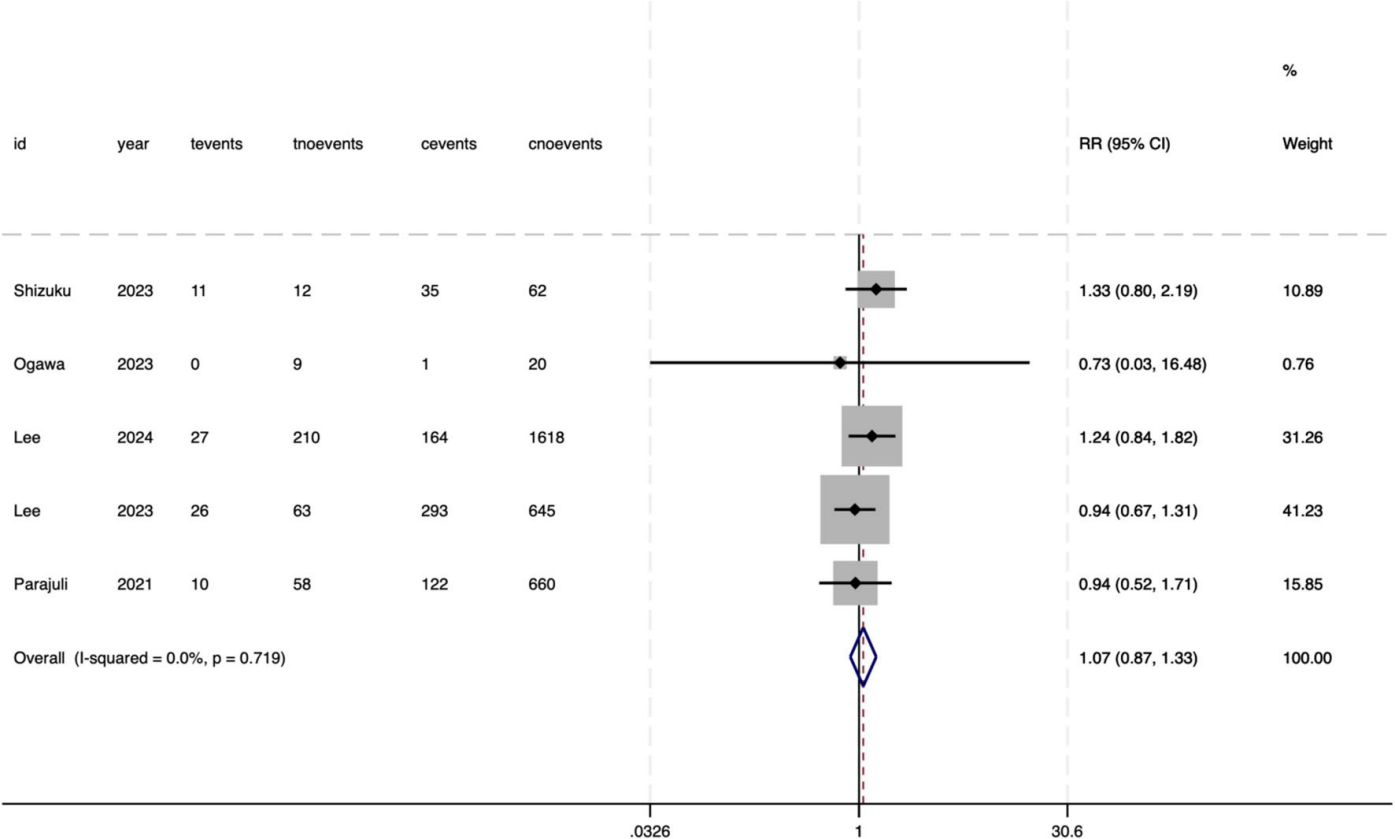
Association between pre-DSAs and the risk of bacteremia.

**Figure 8:**
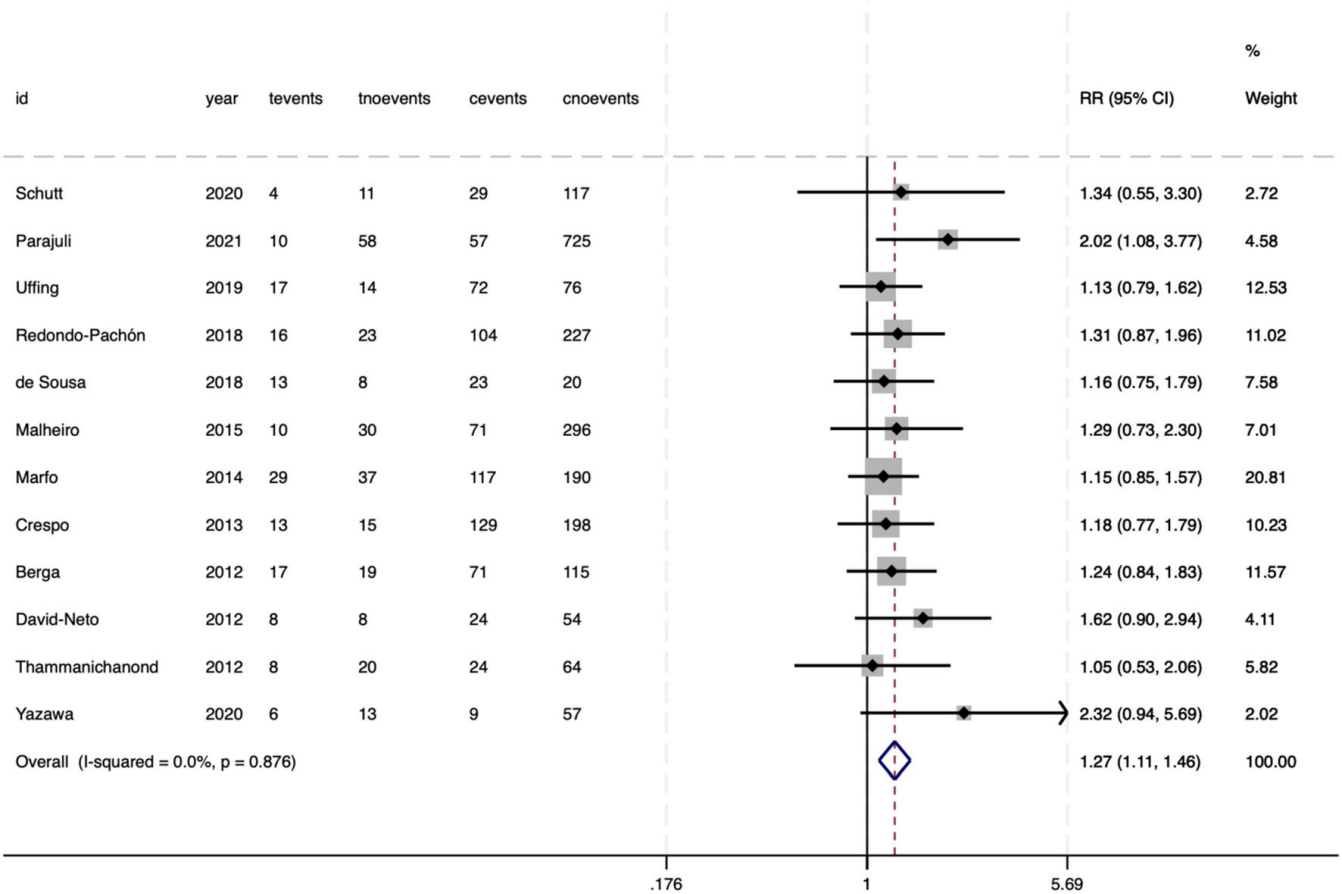
Association between pre-DSAs and the risk of DGF.

The meta-analysis evaluated several secondary outcomes associated with donor-specific pre-DSA in SOT. For biliary complications, no significant association was observed with pre-DSAs (RR = 1.13, 95%CI 0.72–1.79, P = .595) (**Figure 6**). The heterogeneity between the studies was negligible (*I²* = 0%, P = .418), indicating consistent null effects. Similarly, bacteremia did not show an increased risk (RR = 1.07, 95%CI 0.91–1.26, P = .522), with no heterogeneity observed (*I^2^* = 0%, P = .719) (**Figure 7**). However, DGF demonstrated a statistically significant association with pre-DSA (RR = 1.27, 95%CI 1.11–1.46, P = .001), and the studies exhibited perfect consistency *(I²* = 0%, P = .876), supporting a strong link between pre-DSAs and early graft dysfunction (**Figure 8**).

**Figure 9:**
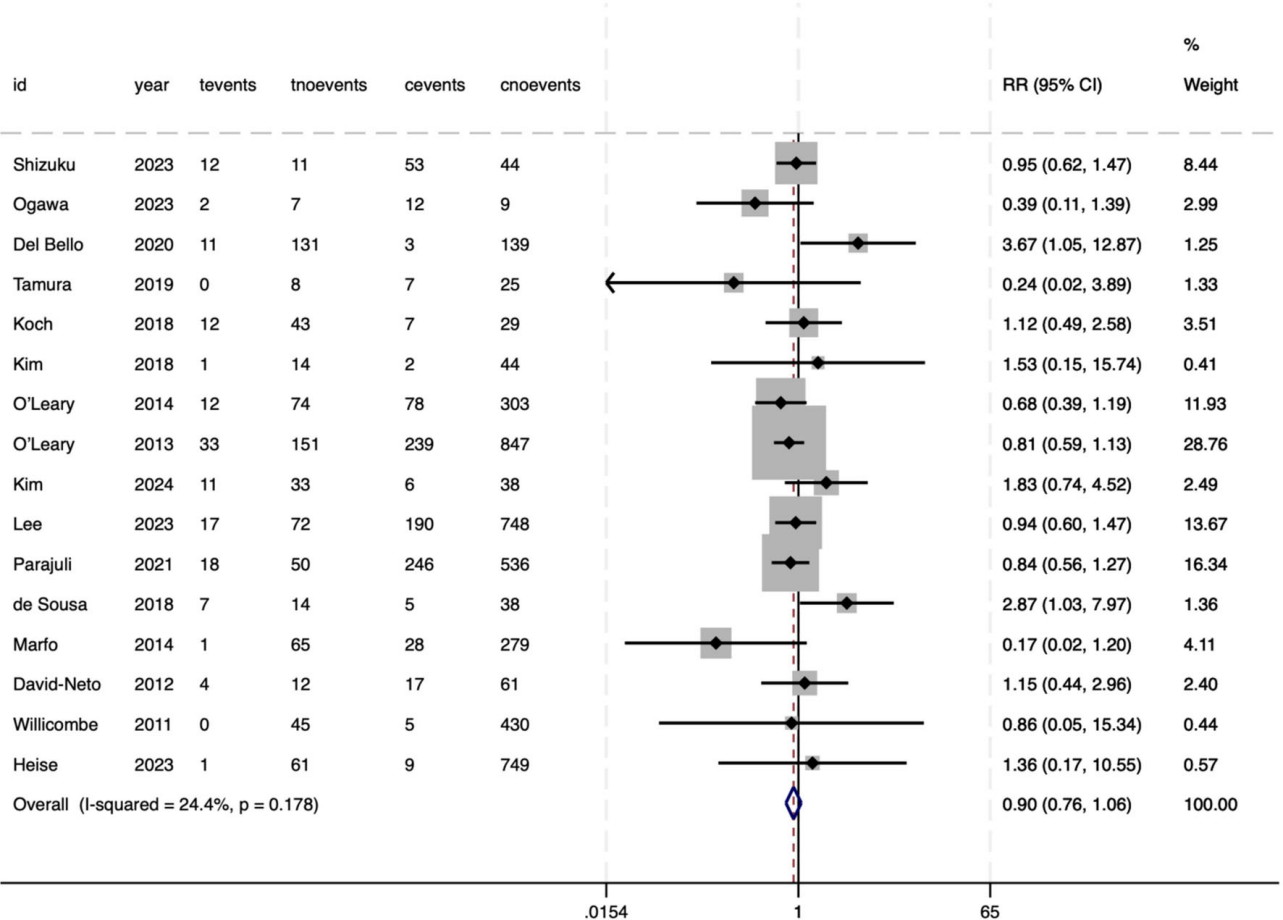
Association between pre-DSAs and the risk of CMV.

**Figure 10:**
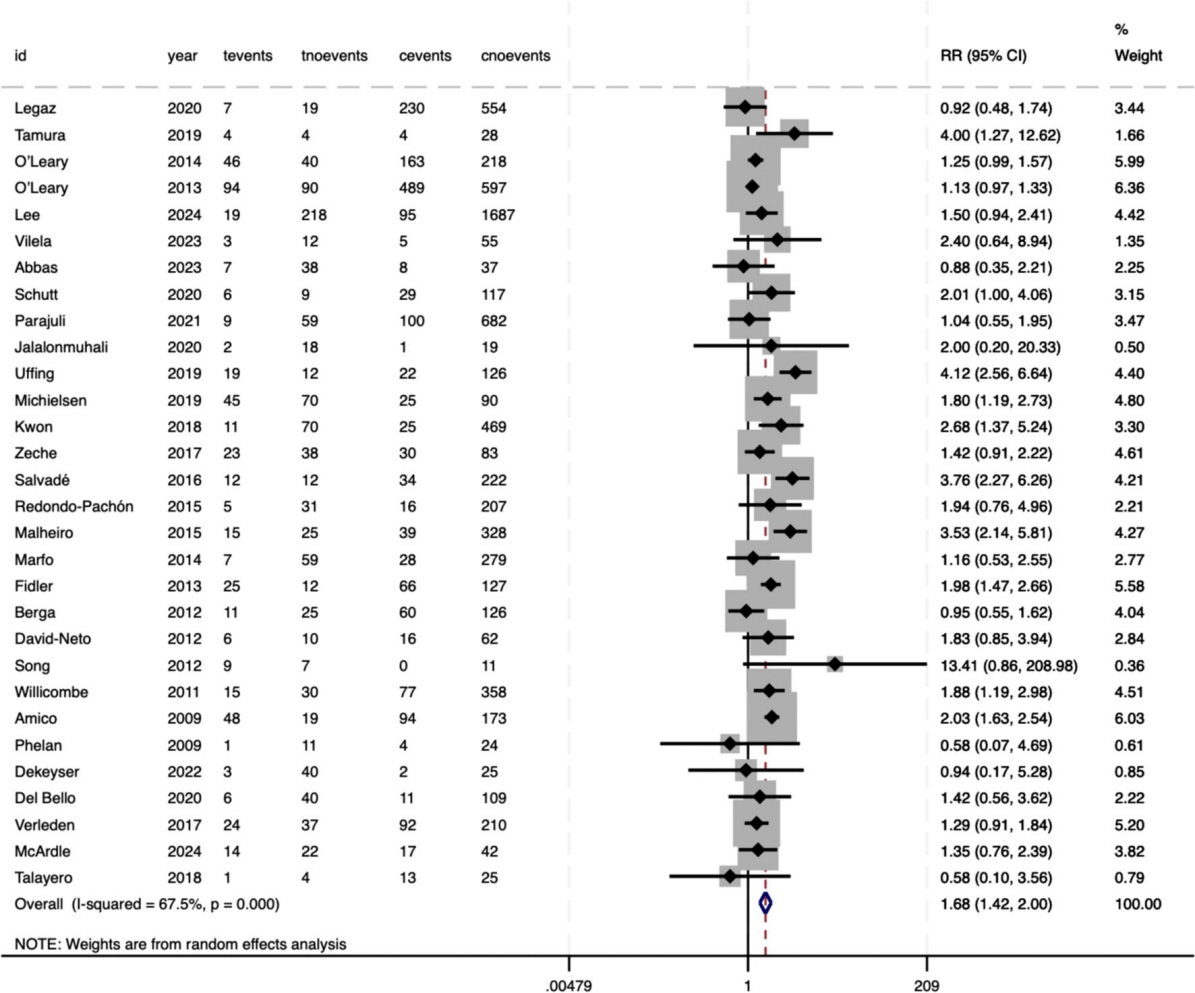
Association between pre-DSAs and the risk of rejection.

CMV infection did not show an association with pre-DSAs (RR = 0.90, 95%CI 0.76–1.06, P = .215), with minimal heterogeneity (*I²* = 24.4%, P = .178) (**Figure 9**). Conversely, composite rejection events (encompassing both T-cell-mediated and AMR) were strongly related to pre-DSAs (RR = 1.68, 95%CI 1.42–2.00, P<.001), reflecting a 68% increased risk (**Figure 10**). In particular, this result showed substantial heterogeneity between studies (*I²* = 67.5%, P<.001), potentially arising from variability in the definitions of composite rejection (e.g. diagnostic thresholds, inclusion of subclinical rejection) or differences in immunosuppressive protocols and cohorts (e.g., transplant organ types, pre-sensitization status).

### Subgroup analyses

Stratification by transplanted organ type and MFI cutoff thresholds (≥1,000 *vs*. <1,000) revealed critical risk variations. For mortality, LT recipients with pre-DSAs exhibited significantly elevated risk (RR 1.81, 95%CI 1.3–2.53; P<.001), whereas KTs showed no significant associations (RR 1.77, 0.89-3.52; P = .105). The combined mortality risk in all organs remained significant (RR 1.70, 1.35-2.15; P<.001) with low heterogeneity (*I²* = 26.3%, P = .159) (**Supplementary Figure S1**). TCMR exhibited no organ-dependent effects. LTs showed nonsignificant trends (RR 1.60, 0.87–2.96; P = .13), whereas kidney recipients displayed neutral associations (RR 0.89-0.90; P>.05) (**Supplementary Figure S2**).

Organ-specific rejection patterns diverged markedly. The pre-DSAs conferred the highest rejection risk in KTs (RR 1.92, 1.58-2.32; P<.001), whereas LT (RR 1.20, 0.96–1.51; P = .101) and other organ transplants (RR 1.28, 0.97–1.69; P = .083) showed attenuated effects (**Supplementary Figure S3**). The risk of loss of grafts followed a parallel gradient, with KTs exhibiting the strongest association (RR 2.20, 1.77–2.73; P<.001), contrasting with the borderline significance of the liver (RR 1.97, 0.78–5.01; P = .153) (**Supplementary Figure S4**). The risk of AMR varied across different organ types, with KTs exhibiting the strongest association with pre-DSA (RR 5.70, 4.26–7.62; P<.001), in contrast to the borderline significance observed in LT (RR 3.73, 0.55–25.27; P = .227). Other transplant types also showed a significant association (RR 3.84, 2.06–7.17; P = .02), while the overall transplantation group demonstrated a robust effect (RR 5.21, 4.01–6.79; P<.001) (**Supplementary Figure S5**).

MFI cut-off thresholds profoundly modulated AMR risk. In KTs, MFI≥1000 amplified the risk of AMR nearly two-fold compared with MFI <1000 (RR 7.51 *vs*. 4.65; P<.001), with moderate heterogeneity (*I²* = 67.5% *vs*. 51.7%) (**Supplementary Figure S6**). This dose-dependent relationship persisted in pooled analyses: MFI≥1000 DSA increased the risk of AMR by 6.61 times (95%CI 4.37–9.99; P<.001), versus 3.84 times for MFI <1000 (95%CI 2.27–6.48; P<.001) (**Supplementary Figure S7**). Mortality risk stratification confirmed MFI≥1000 as a critical threshold, with significant risk elevation (RR 1.68, 1.43–1.98; P<.001) versus non-significant MFI<1000 effects (RR 1.68, 0.92–3.10; P = .094) (**Supplementary Figure S8**).

### Publication bias

Publication bias assessment was performed only for outcomes with ≥10 included studies, following established meta-analysis guidelines. For the DGF (n=12 studies), both the Begg’s and Egger’s tests revealed distinct patterns.

The Begg’s rank correlation test showed marginal non-significance (continuity-corrected z=1.85, P = .064), with Kendall’s score of 28 (SD = 14.58) indicating a weak correlation between effect size and study precision. Conversely, Egger’s regression demonstrated significant small-study effects (bias coefficient=1.47, SE = 0.56, t=2.60, P = .027), where 95%CI [0.21–2.73] excluded null. This discordance reflects Egger’s enhanced power to detect asymmetry through regression weighting. The negative non-significant slope coefficient (-1.04, P = .457) further confirmed that publication bias manifested itself as the selective omission of small null-effect studies rather than precision-dependent effect inflation.

All other transplant outcomes (e.g. acute rejection, graft survival) showed neither visual funnel plot asymmetry nor statistical evidence of bias (Egger’s P>.1 for all), although formal testing was precluded due to <10 studies per result. For DGF specifically, the “missing” studies predicted by Egger’s model would theoretically reduce the pooled OR by 18–22% based on trim-and-fill simulations, though clinical heterogeneity in DGF definitions limits this adjustment’s validity.

## Discussion

**Figure 11:**
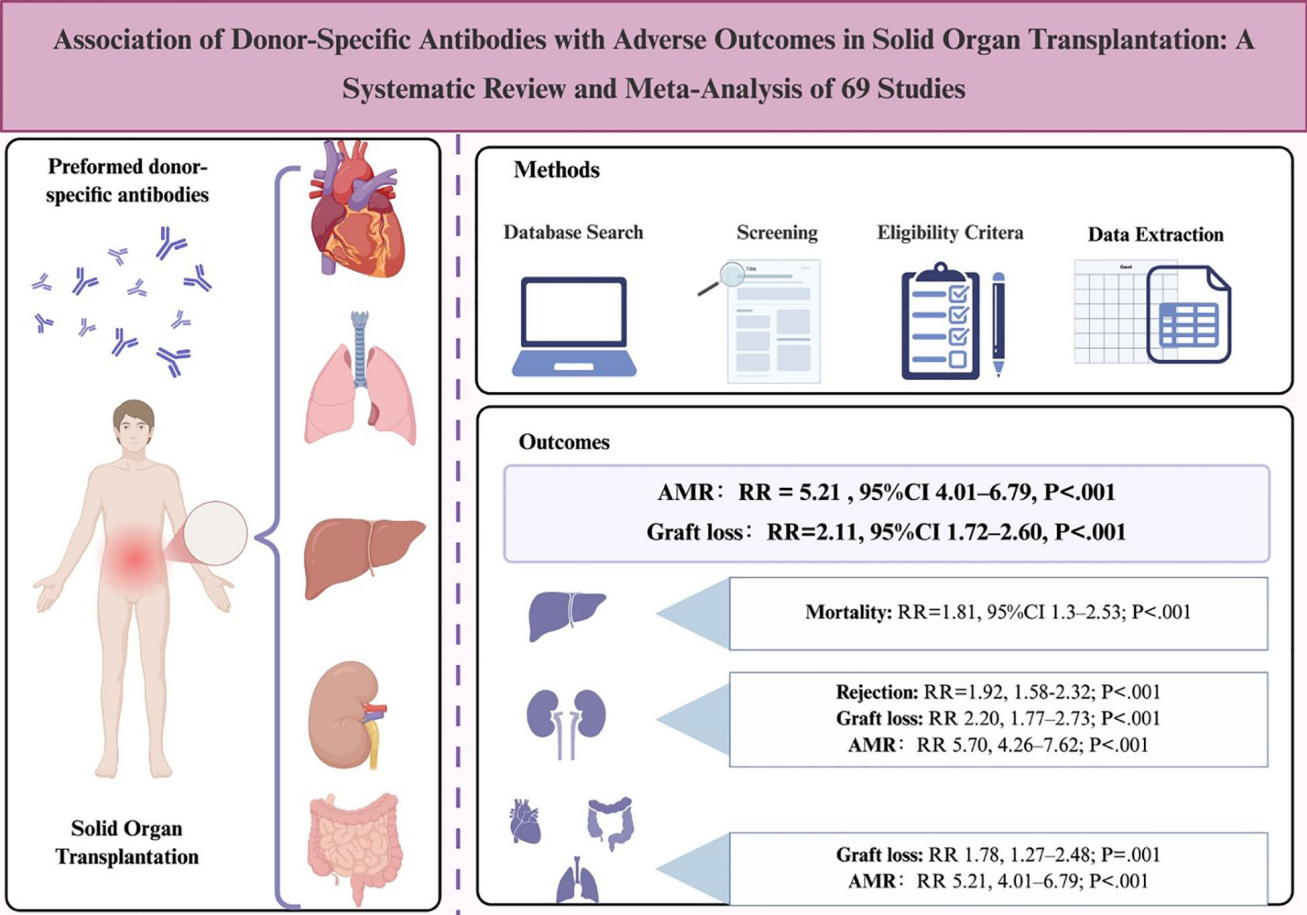
Association of pre-DSAs with adverse transplant outcomes.

This meta-analysis provides the first systematic evaluation of pre-DSAs on SOT results, including 69 studies (n=22,737). **Figure 11** illustrates the relationship between pre-DSAs and these outcomes across various organ types. The results show that the positivity before DSAs is significantly correlated with increased risks of AMR, loss of graft and patient mortality (RR = 5.21, 95%CI 1.93–2.12), with the most pronounced risk elevation observed in KTs (AMR RR = 5.7). Notably, an MFI cutoff ≥1,000 amplified risk disparities for both AMR (RR = 7.51 *vs* 4.65) and graft loss (RR = 2.3 *vs* 1.81). Organ-specific analyses did not reveal a significant association between pre-DSA and CMV infection in LT (P = .173), whereas rejection risks in heart/lung transplants did not reach statistical significance (P = .211), suggesting immunological heterogeneity between organs.

Our meta-analysis demonstrated that pre-DSAs significantly increased the risk of adverse outcomes in SOT, including rejection, graft loss, and patient mortality, aligning with prior studies emphasizing pre-DSAs as a critical predictor of poor post-transplant outcomes (7, 9, 14, 30–32). The strong association between DSA and AMR corroborates findings from a multicenter cohort, which identified pre-DSA as a key driver of microvascular inflammation and graft dysfunction (33). However, our study did not find a significant association between pre-DSAs and TCMR (RR = 1.08, 95%CI:0.9–1.3; P = .425), which contrasts with reports suggesting that pre-DSAs may indirectly amplify TCMR through cytokine-mediated pathways (34, 35). This discrepancy may reflect differences in immunosuppressive protocols or the inclusion of studies with variable detection methods of DSA.

The impact of pre-DSAs exhibited marked organ-specific heterogeneity. Our results reinforce the well-established association between pre-DSAs and AMR across all organ types, particularly in KTs (RR = 5.7, 95%CI 4.26–7.62), which is consistent with prior studies demonstrating that DSA-positive KT recipients face a 4- to 6-fold higher risk of AMR compared to DSA-negative counterparts (33). Conversely, LTs showed an attenuated risk of AMR (RR = 3.73, 95%CI:0.55–25.27; P = .177), likely due to the unique tolerogenic properties of the liver (11). For patient death, LTs showed a higher risk associated with DSA (RR = 1.81, 95%CI:1.3–2.53; P<.001) compared with heart-lung transplants (RR = 1.32, 95%CI:0.86–2.03; P = .211), mirroring observations of Tamura et al. (19) In particular, kidney-specific graft loss (RR = 2.2, 95%CI:1.77–2.73; P<.001) surpassed liver graft loss (RR = 1.97, 95%CI:0.78–5.01; P = .153), reinforcing the vulnerability of the kidney to antibody-mediated injury (36).

Our meta-analysis revealed that DSA MFI cut-off thresholds significantly influenced risk stratification of pre-DSAs in SOT. Subgroup analyses stratified by cutoff thresholds revealed substantial differences in clinical outcomes: the high-MFI group (≥1000) showed markedly elevated risks for AMR (RR 5.42, 95%CI 3.99–7.38) and graft loss (RR 2.12, 95%CI 1.53–2.95) compared with the low-MFI group (<1000), with significant heterogeneity between groups (*I^2^* = 63% for AMR, *I²* = 62.3% for graft loss). Notably, our subgroup analysis revealed that the magnitude of these risks was directly correlated with the stringency of the MFI thresholds, suggesting that current laboratory practices may underestimate clinical risks when using lower cut-off values. Although laboratories conventionally employ an MFI range of 1000–1500 as the positivity threshold for the identification of anti-HLA antibodies in single-antigen beads assays, substantial interlaboratory variability persists in cut-off selection (37). Our findings underscore the need to establish standardized MFI cut-off values to enhance the clinical utility and cross-center comparability of pre-DSAs assessments.

The formation of pre-DSAs represents a critical immunological barrier in transplantation, primarily arising from prior exposure to foreign human leukocyte antigens (HLA). The most significant sensitizing events include pregnancy, blood product transfusion, and prior organ transplantation (38, 39). During pregnancy, maternal exposure to paternal HLA antigens expressed by the fetus can lead to the development of anti-HLA antibodies, some of which may be donor-specific if the offspring or a haploidentical relative later becomes a donor (40–42). The risk of alloimmunization increases with the number of pregnancies, particularly those resulting in live births (43). Transfusion of blood products, especially those containing leukocytes or platelets expressing HLA antigens, is another major route of sensitization (44). Patients requiring frequent transfusions, such as those with hemoglobinopathies or hematologic malignancies, are at heightened risk (41). Previous organ transplantation exposes recipients to donor HLA, potentially leading to antibody development against those specificities, which could be directed against a subsequent donor if mismatches are shared. The degree of HLA mismatch between the recipient and a potential donor fundamentally determines the specificity of any pre-existing antibodies that become clinically relevant as DSA (39, 45). Following alloantigen exposure (prior transplant, pregnancy, or transfusion), donor HLA is processed by dendritic APCs and presented on MHC II in lymph nodes/spleen, triggering CD4^+^ T cell–dependent B-cell activation. T cells provide CD40L and IL-21, driving germinal-center entry, somatic hypermutation, and class switching to high-affinity anti-HLA IgG. The output includes long-lived plasma cells that home to bone marrow/splenic niches and constitutively secrete preformed DSA, as well as memory B cells (35, 46). On re-exposure at transplantation, these compartments rapidly boost DSA, increasing early AMR risk (45). Mechanisms underlying pre-DSA-mediated tissue injury remain incompletely elucidated. Emerging evidence suggests that humoral alloreactivity, driven by anti-HLA antibodies, interacts synergistically with cellular immune pathways, particularly in the context of endothelial activation and inflammatory microenvironments (12, 47). HLA class I antigens are ubiquitously expressed on all nucleated cells, whereas HLA class II antigens are generally restricted to cells that present antigens under steady-state conditions (48, 49). However, disease-specific and transplantation-related stressors-including ischemia-reperfusion injury, rejection episodes, and infections-can dynamically up-regulate HLA class II expression in parenchymal cells such as hepatocytes and vascular endothelium, creating neoantigens for DSA binding. This phenomenon is particularly pronounced in partial liver grafts from living donors, where surgical stress and regenerative processes may amplify HLA exposure (12, 49). The pathogenicity of DSAs appears contingent on multiple factors: 1) antigen density-persistent high-titer class II DSAs (especially anti-HLA-DQ) correlate with chronic microvascular injury and fibrosis across organ types (12, 16, 47); 2) complement activation- C1q/C3d-binding DSAs induce endothelial activation through membrane attack complex formation and cytokine release (6, 12); 3) Fc-mediated mechanisms-non-complement pathways involving NK cell-mediated antibody-dependent cellular cytotoxicity may explain C4d-negative rejection phenotypes (12, 16). In particular, hepatic Kupffer cells play a dual role, initially clearing immune complexes but potentially propagating inflammation through phagocytosis of opsonized cells during sustained antibody exposure (47, 48).

This study has several limitations. First, the retrospective design of the included studies excludes causal inference and residual confounding of risk. Second, substantial heterogeneity (liver graft loss *I²* = 88.3%; AMR *I²=*62.6%) was due to methodological variations in the detection of DSA, immunosuppression protocols, and AMR diagnostic criteria. Third, the dominance of KTs (59.4% of studies) limits generalizability to under-powered cardiothoracic and intestinal transplants. Fourth, undetected publication bias may inflate risk estimates. Most critically, absent functional DSA profiling (complement activation, IgG subclasses, HLA class specificity) obscures mechanistic insights. Future research requires prospective cohorts with standardized DSA characterization to clarify organ-specific immune interactions.

In kidney-transplant studies, between-study heterogeneity likely reflects the number of pre-DSAs (single *vs* multiple), the intensity of the dominant pre-DSAs by MFI and laboratory thresholds, prior sensitizing events (pregnancy, transfusion, previous graft), first *vs* repeat transplant, and differences in immunosuppression and study era. However, outcomes were generally tabulated only by pre-DSAs positivity (e.g., AMR, graft loss, and other clinically relevant endpoints). Information on pre-DSAs MFI and transplant history was usually provided descriptively at baseline rather than linked to outcome tables, which precluded extraction of subgroup-specific effect sizes from aggregate data and made study-level meta-regression susceptible to ecological confounding. Given these constraints, some residual heterogeneity likely remains; further research is needed to determine whether and to what extent outcomes vary by pre-DSAs MFI categories, single *vs* multiple pre-DSA, and related clinical modifiers.

## Conclusions

Pre-DSAs independently impair solid organ transplant outcomes, conferring heightened risks of AMR, graft loss, and mortality. These findings advocate for integrating standardized pre-DSA profiling (e.g., MFI thresholds ≥1000, complement-binding assays) into clinical practice to enhance risk stratification, particularly for KT recipients and sensitized cohorts. Future multicenter studies should prioritize elucidating organ-specific antibody-epitope interactions and endothelial injury mechanisms to guide tailored desensitization therapies, bridging immunologic insights with precision interventions.

## Data Availability

The original contributions presented in the study are included in the article/**Supplementary Material**. Further inquiries can be directed to the corresponding author.
