# Supplementary material for: Association of donor-specific antibodies with adverse outcomes in solid organ transplantation: A systematic review and meta-analysis of 69 studies
Source: Front Immunol. 2025 Sep 12;16:1633853. doi: 10.3389/fimmu.2025.1633853 (PMC12463609; doi:10.3389/fimmu.2025.1633853)
Supplement: Supplementary file 1 [file DataSheet1.docx]

S1-table : Search Strategy

| Search number | Query | Results |
| --- | --- | --- |
| 4 | ((((((((((((((((((liver transplantation[Title/Abstract]) OR (liver transplant[Title/Abstract])) OR (liver-kidney transplantation[Title/Abstract])) OR (kidney transplantation[Title/Abstract])) OR (kidney transplant[Title/Abstract])) OR (renal transplant[Title/Abstract])) OR (renal transplantation[Title/Abstract])) OR (kidney graft[Title/Abstract])) OR (lung transplantation[Title/Abstract])) OR (lung transplant[Title/Abstract])) OR (heart transplantation[Title/Abstract])) OR (heart transplant[Title/Abstract])) OR (cardiac transplantation[Title/Abstract])) OR (intestinal transplantation[Title/Abstract])) OR (intestinal[Title/Abstract] AND multivisceral transplantation[Title/Abstract])) OR (visceral transplantation[Title/Abstract])) OR (organ allocation[Title/Abstract])) AND ((((((((DSA[Title/Abstract]) OR (DSAs[Title/Abstract])) OR (pfDSA[Title/Abstract])) OR (pDSA[Title/Abstract])) OR (HLA DSA[Title/Abstract])) OR (HLA-DSA[Title/Abstract])) OR (donor-specific antibodies[Title/Abstract])) OR (donor-specific HLA antibodies[Title/Abstract]))) AND (((((preformed[Title/Abstract]) OR (pre-transplant[Title/Abstract])) OR (pre-existing[Title/Abstract])) OR (pretransplant[Title/Abstract])) OR (pretransplantation[Title/Abstract])) | 717 |
| 3 | ((((preformed[Title/Abstract]) OR (pre-transplant[Title/Abstract])) OR (pre-existing[Title/Abstract])) OR (pretransplant[Title/Abstract])) OR (pretransplantation[Title/Abstract]) | 83983 |
| 2 | (((((((DSA[Title/Abstract]) OR (DSAs[Title/Abstract])) OR (pfDSA[Title/Abstract])) OR (pDSA[Title/Abstract])) OR (HLA DSA[Title/Abstract])) OR (HLA-DSA[Title/Abstract])) OR (donor-specific antibodies[Title/Abstract])) OR (donor-specific HLA antibodies[Title/Abstract]) | 14512 |
| 1 | ((((((((((((((((liver transplantation[Title/Abstract]) OR (liver transplant[Title/Abstract])) OR (liver-kidney transplantation[Title/Abstract])) OR (kidney transplantation[Title/Abstract])) OR (kidney transplant[Title/Abstract])) OR (renal transplant[Title/Abstract])) OR (renal transplantation[Title/Abstract])) OR (kidney graft[Title/Abstract])) OR (lung transplantation[Title/Abstract])) OR (lung transplant[Title/Abstract])) OR (heart transplantation[Title/Abstract])) OR (heart transplant[Title/Abstract])) OR (cardiac transplantation[Title/Abstract])) OR (intestinal transplantation[Title/Abstract])) OR (intestinal[Title/Abstract] AND multivisceral transplantation[Title/Abstract])) OR (visceral transplantation[Title/Abstract])) OR (organ allocation[Title/Abstract]) | 224231 |

Supplementary Figures


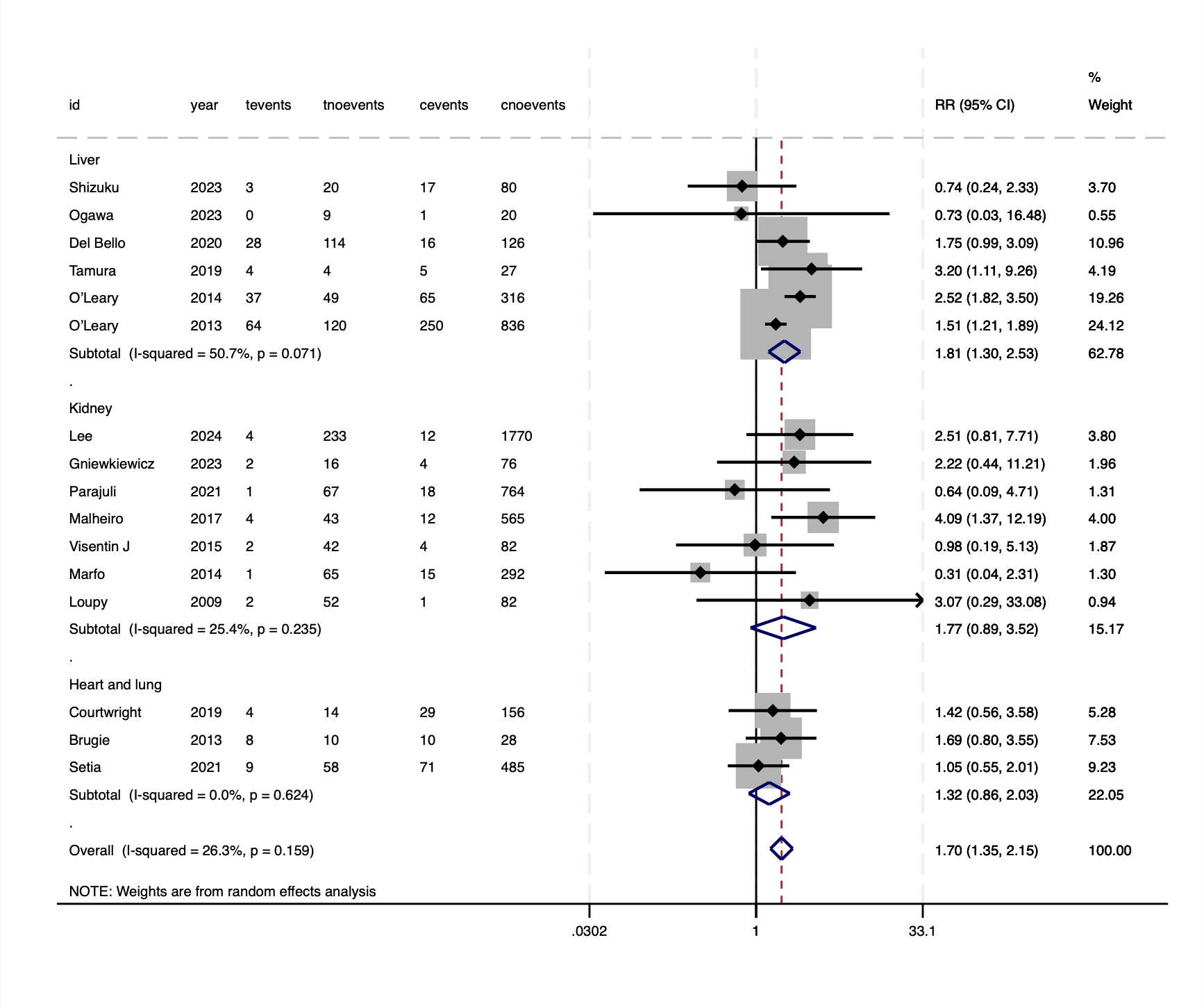


S1 Figure.Patient death.


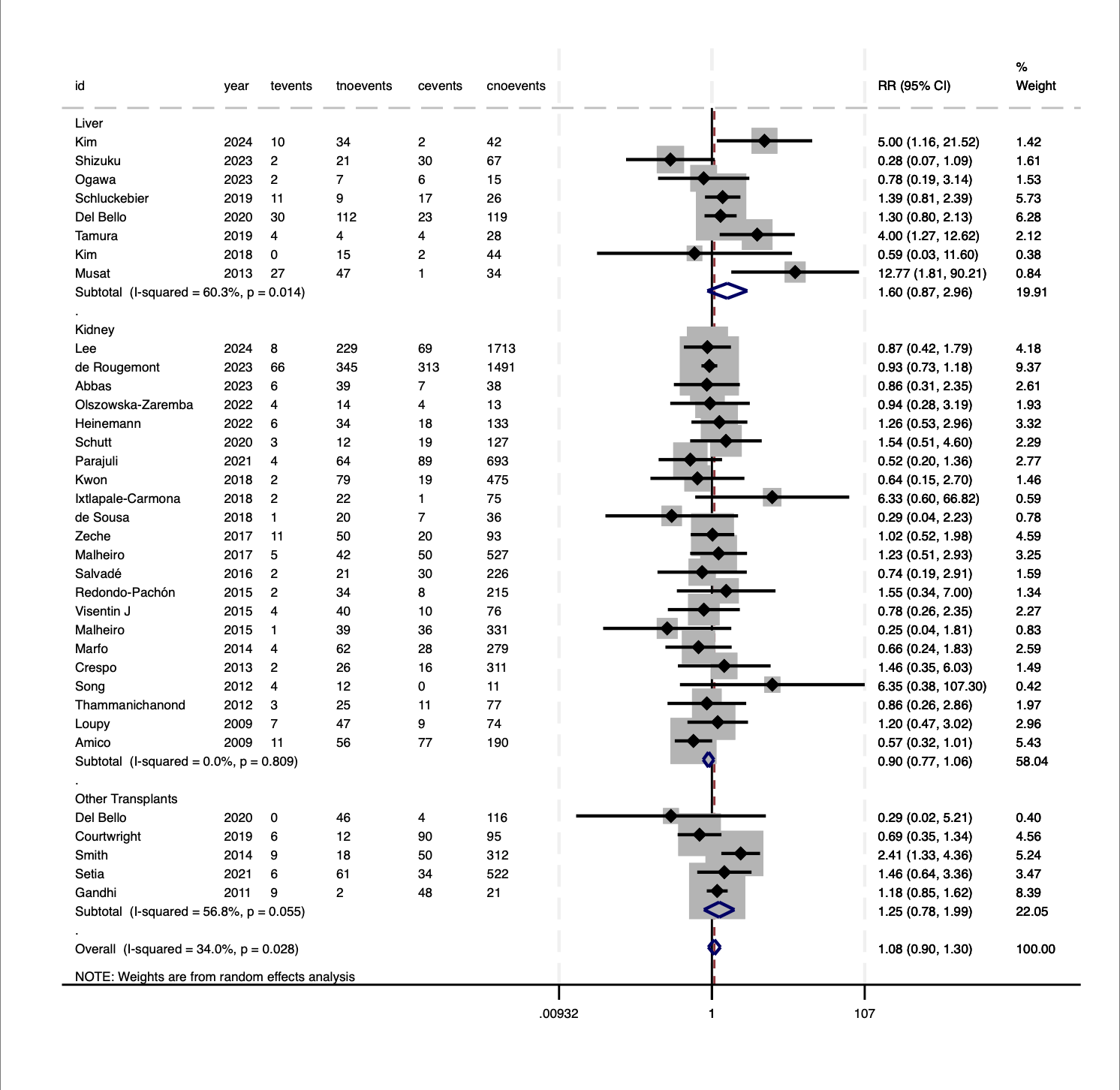


S2 Figure. TCMR.


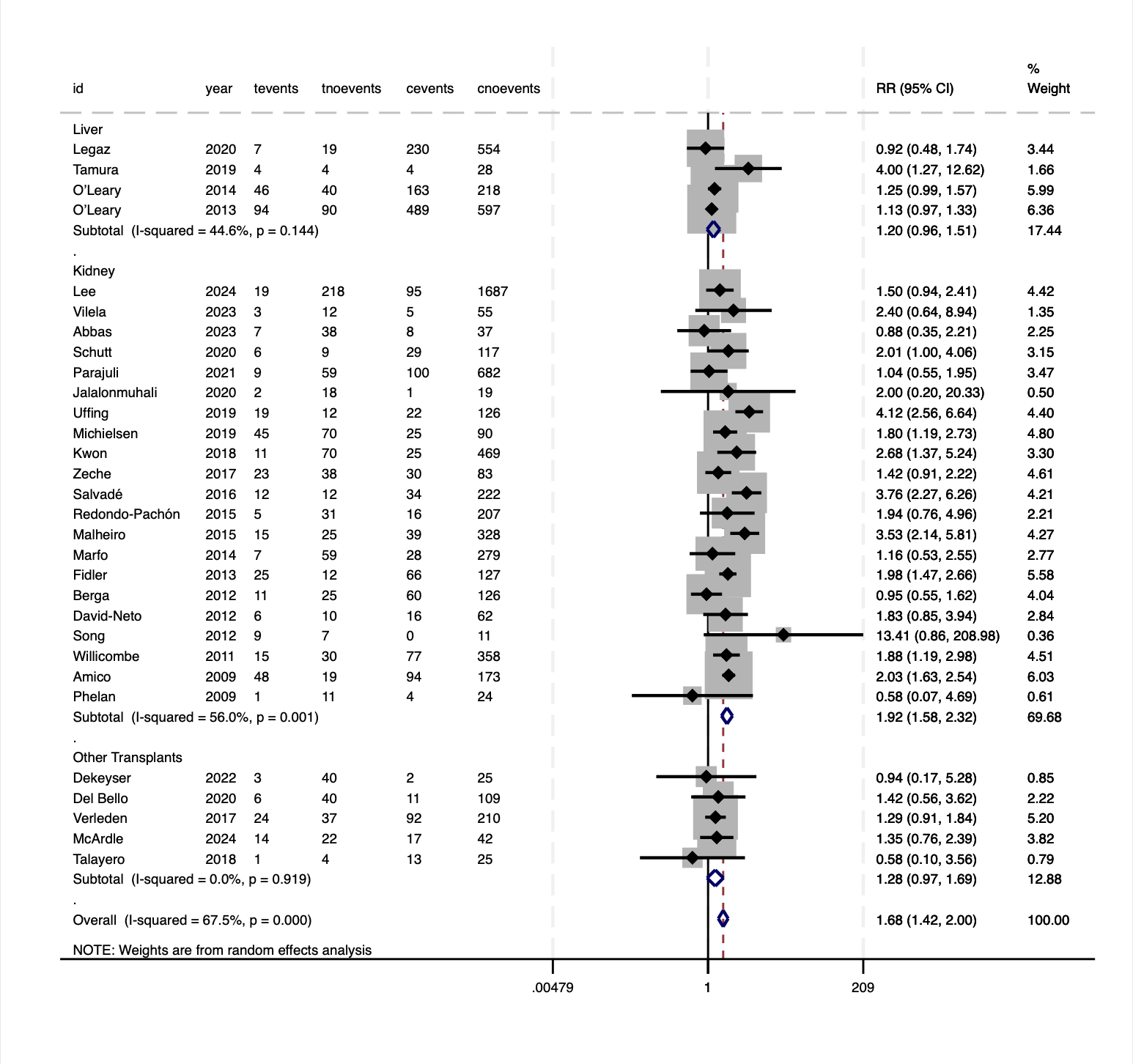


S3 Figure.Rejection.


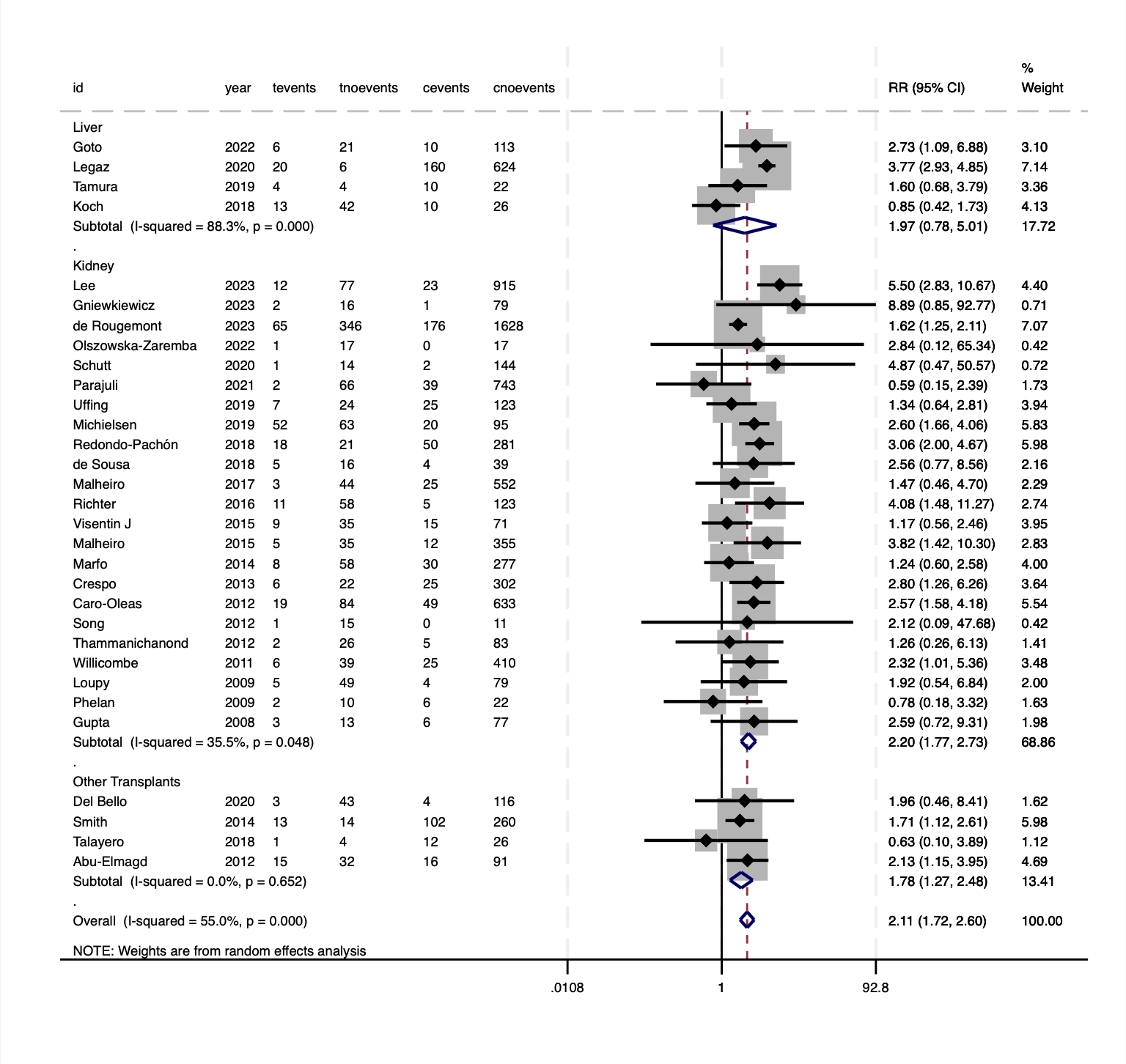


S4 Figure.Graft loss.


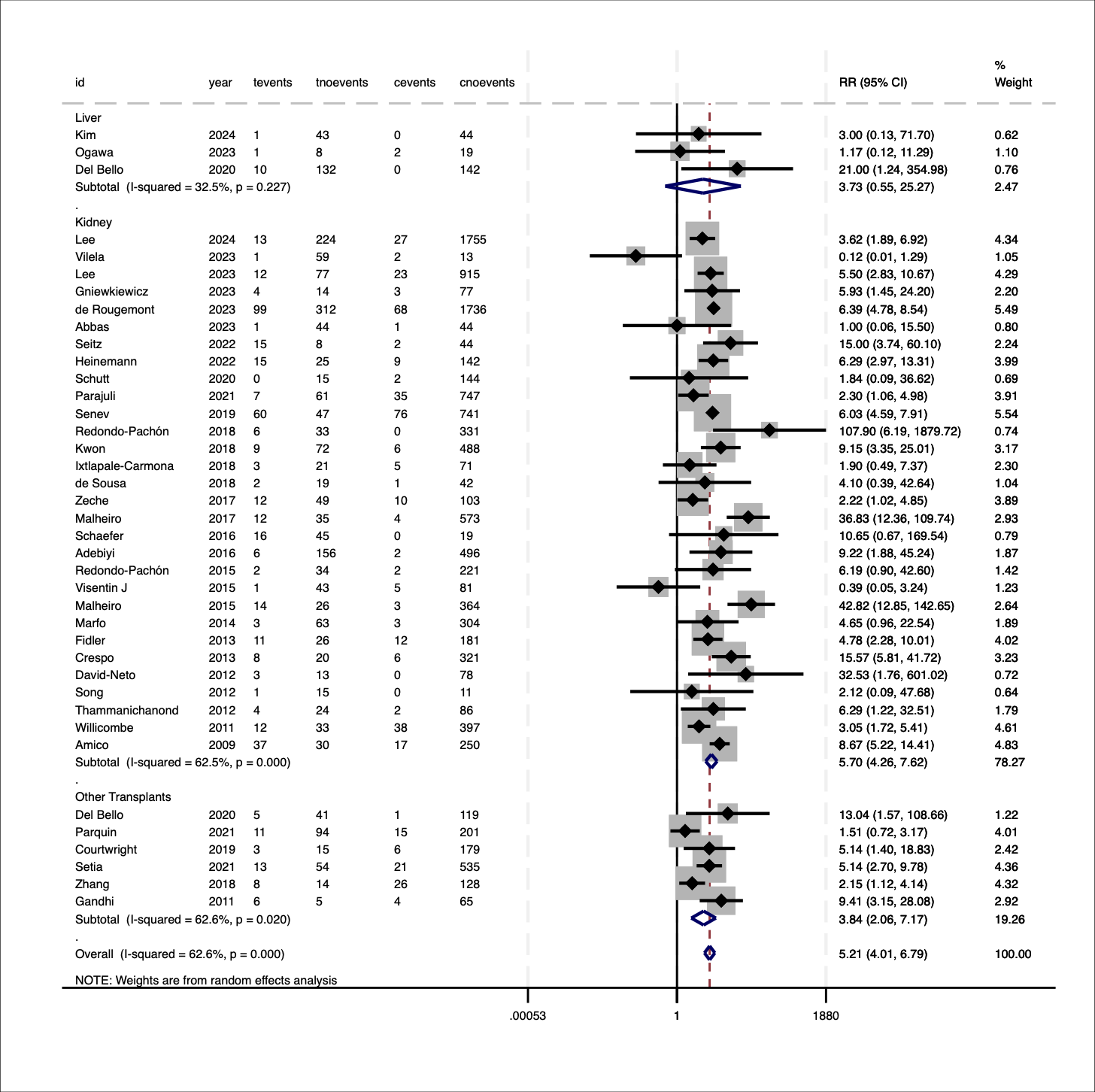


S5 Figure.AMR.


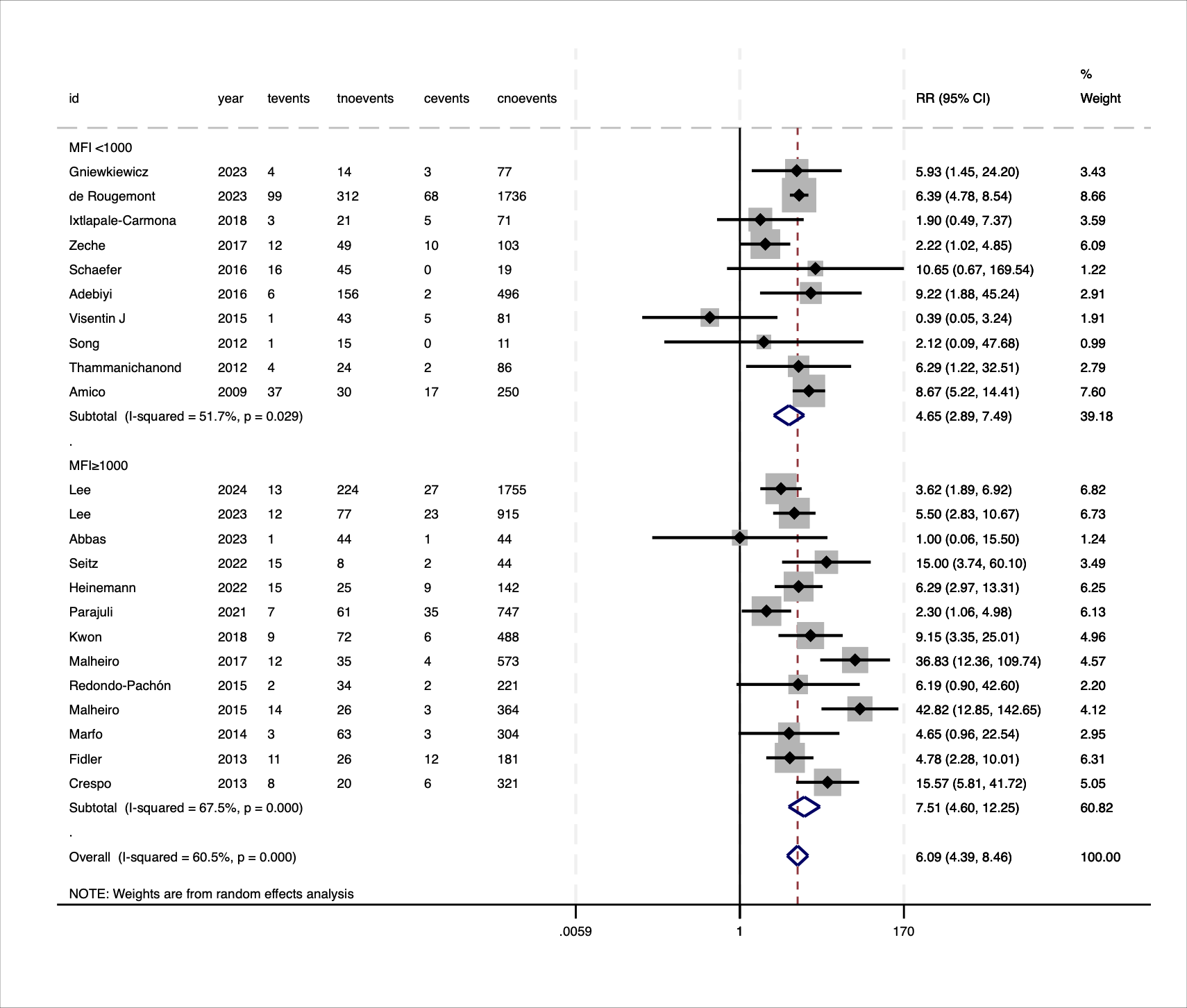


S6 Figure. AMR.


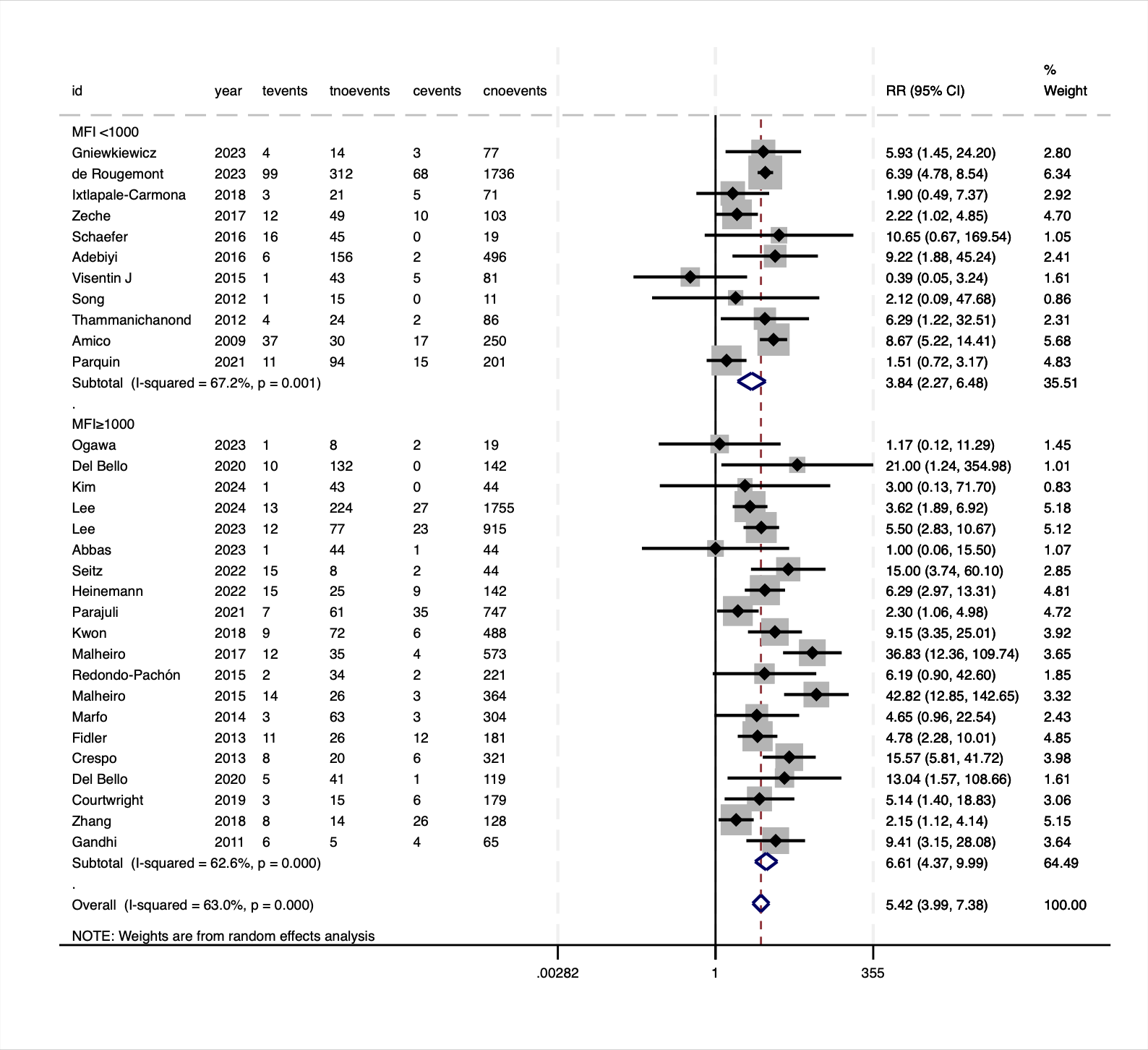


S7 Figure. AMR.


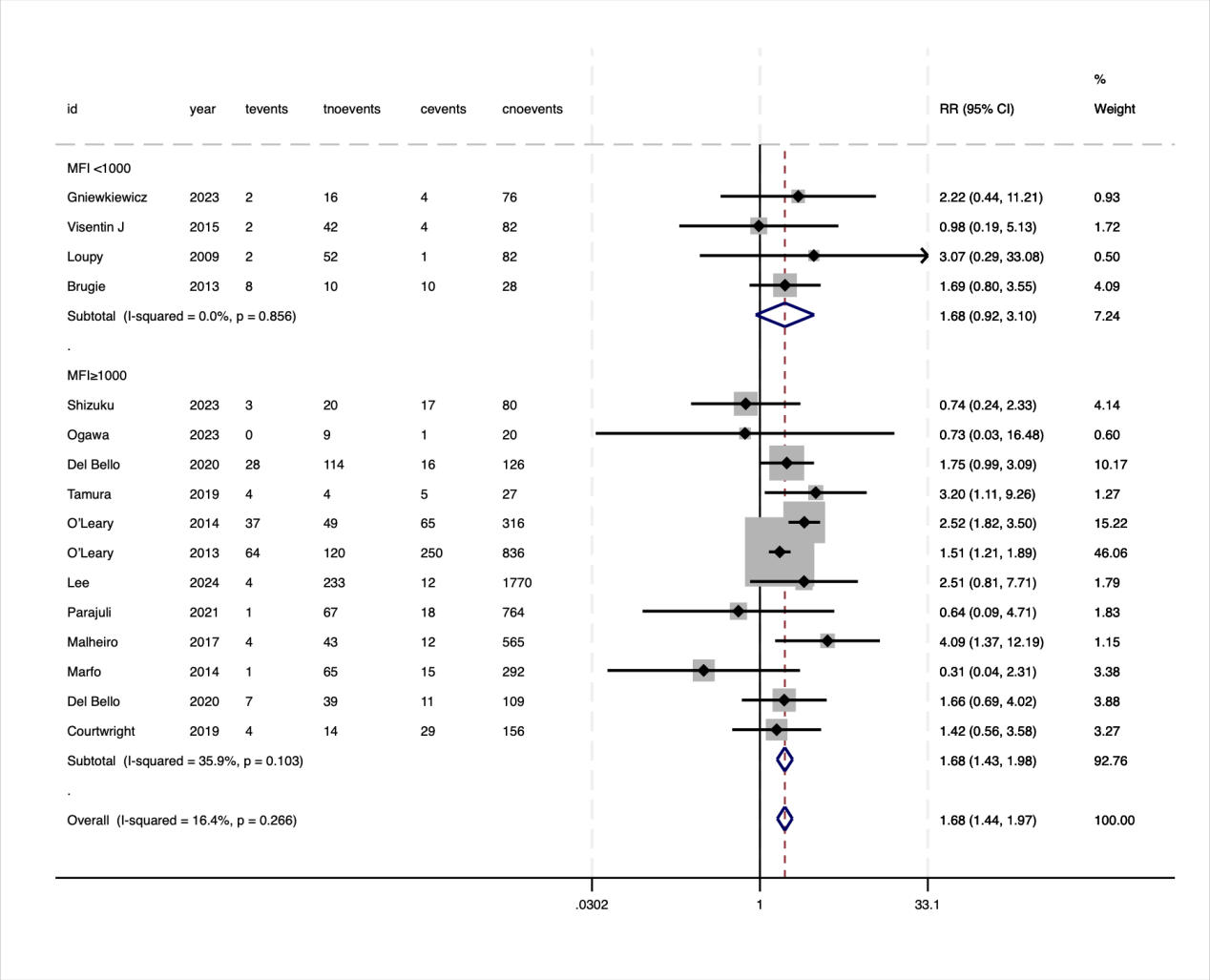


S8 Figure. Patient death.
